# Double Trouble at High Density: Cross-Level Test of Resource-Related Adaptive Plasticity and Crowding-Related Fitness

**DOI:** 10.1371/journal.pone.0091503

**Published:** 2014-03-13

**Authors:** André Gergs, Thomas G. Preuss, Annemette Palmqvist

**Affiliations:** 1 Department of Environmental, Social and Spatial Change, Roskilde University, Roskilde, Denmark; 2 Institute for Environmental Research, RWTH Aachen University, Aachen, Germany; Texas Tech University, United States of America

## Abstract

Population size is often regulated by negative feedback between population density and individual fitness. At high population densities, animals run into double trouble: they might concurrently suffer from overexploitation of resources and also from negative interference among individuals regardless of resource availability, referred to as crowding. Animals are able to adapt to resource shortages by exhibiting a repertoire of life history and physiological plasticities. In addition to resource-related plasticity, crowding might lead to reduced fitness, with consequences for individual life history. We explored how different mechanisms behind resource-related plasticity and crowding-related fitness act independently or together, using the water flea *Daphnia magna* as a case study. For testing hypotheses related to mechanisms of plasticity and crowding stress across different biological levels, we used an individual-based population model that is based on dynamic energy budget theory. Each of the hypotheses, represented by a sub-model, is based on specific assumptions on how the uptake and allocation of energy are altered under conditions of resource shortage or crowding. For cross-level testing of different hypotheses, we explored how well the sub-models fit individual level data and also how well they predict population dynamics under different conditions of resource availability. Only operating resource-related and crowding-related hypotheses together enabled accurate model predictions of *D. magna* population dynamics and size structure. Whereas this study showed that various mechanisms might play a role in the negative feedback between population density and individual life history, it also indicated that different density levels might instigate the onset of the different mechanisms. This study provides an example of how the integration of dynamic energy budget theory and individual-based modelling can facilitate the exploration of mechanisms behind the regulation of population size. Such understanding is important for assessment, management and the conservation of populations and thereby biodiversity in ecosystems.

## Introduction

Population regulation is key to the understanding of evolutionary processes, ecosystem health and biodiversity. Population dynamics can depend on the fitness of individual population members, and in turn, individual fitness can depend on population density [1]. This negative feedback between population density and individual fitness might have several causes that can act simultaneously within a population [2]. At high population densities, animals basically encounter double trouble: they might suffer from intraspecific competition for resources and not considering space as a resource, from negative interference between individuals regardless of resource availability, referred to as crowding. Competition, together with overexploitation of resources, often results in reduced individual growth, fecundity and survival, thereby limiting population density [3]. If delayed feedback processes are involved, this resource-related mechanism might lead to the periodic fluctuations in population density often observed in natural and laboratory systems [4], [5]. Resource limitation as such is suggested to be a primary driver of the manifestation of trade-offs between life history traits [6], [7], which might constrain the evolution of these traits.

Individual organisms are able to adapt to resource shortage and to some extent resist competition-induced starvation by exhibiting a repertoire of life history or physiological plasticities. This might include variations in traits such as offspring size [8], [9], [10], morphological changes [11], and the reduction of energy expenditure or changes in energy allocation [12]. Similarly, crowding can lead to reductions in somatic growth [13], increased mortality [14] and reduced larval fitness [15]. Alterations in crowding-related fitness can be a result of direct interference among individuals [16] or chemicals released by individuals [17], [18]. Crowding-mediated density-dependence has been suggested to play an important role in stabilising population dynamics in enriched environments [19] and in population responses to abiotic stressors [20], [21].

Resource-related and crowding-related mechanisms have usually been investigated separately. However, elucidating how different mechanisms act at the level of individual organisms and how mechanisms interact at the population level is essential for understanding the regulation of population size and for predicting population responses to anthropogenic alterations in the environment. Here, we use the water flea *Daphnia magna* as a case study to explore how resource-related adaptive plasticity and crowding-related fitness operate independently or together across different biological levels, i.e. individual organisms and populations. *Daphnia* species are widely distributed in all (freshwater) parts of the world. Due to their filter-feeding efficiency and their role as a food source for other invertebrates and fish, daphnids are important components of freshwater food-webs [22], [23]. Moreover, the genus of *Daphnia* is one of the best-studied taxa in ecology [4], [24] and is sensitive to a wide range of chemicals, making them important test organisms in ecotoxicology [25]. To cope with conditions of low resource availability, daphnids can increase their feeding rates [11] or change life history strategies as a result of changing energy allocation within the body [26]. Isolated daphnid individuals were observed to change their filtering limb beat rate [27] and the size of filtering limbs [28] in response to food shortage. This immediate response might be followed by a slower morphological response within a population resulting from the replacement of individuals [28]. For crowding, the presence of conspecifics was found to depress feeding and (possibly as a result) to reduce growth and reproduction [17], [18], [29], [30]. Crowding-related behavioural changes and morphological alterations, such as reduced tail spine length and increased mortality have also been reported [30], [31]. In *D. magna*, crowding was found to induce a life-strategy shift by intra-specific interaction [32]: at high densities and sufficient food supply, daphnids produced fewer but larger neonates compared to non-crowded mothers; these larger neonates showed higher individual fitness, i.e. they contained more lipids and were able to resist starvation to a greater extent than offspring produced in non-crowded controls. Based on their observation of multiple generations, Cleuvers et al. [32] proposed high population density as a proximate factor mediating life history alterations observed at conditions of resource limitation.

In this study, we focused on six hypotheses concerning how resource-related adaptive plasticity and crowding can affect individual life history and population dynamics. In response to resource shortage, daphnids might: 1) increase their filtration rate, 2) change energy allocation within the body or 3) adapt both filtration rate and energy allocation. Under crowding conditions, an individual’s fitness might be changed due to 4) feeding interference resulting in decreased filtration rate, 5) increased maintenance costs as a result of density stress, behavioural or morphological changes or 6) a combination of decreased filtration and increased reproductive costs, e.g. due to the production of larger eggs.

Empirically exploring how these different resource- and crowding-related mechanisms affect individual life history, following resource allocation within an organism and testing the significance of isolated mechanisms at the population level is difficult, if not impossible. For extrapolating population-level consequences from individual-level life history variations and for testing different hypotheses, we therefore used an individual-based model (IBM) where *Daphnia magna* individuals are represented by formulations provided by dynamic energy budget (DEB) theory [12]. This theory provides first-principle rules for the uptake and utilisation of energy and is, due to widespread empirical patterns in metabolic behaviour among many different organisms, expected to cover basically all species [33]. Dynamic energy budget models are particularly suitable to follow the uptake of food and the assimilation of energy into the reserve, which in turn are used for the maintenance, growth and reproduction of individual organisms [34], and provide a solid basis for cross organisational-level extrapolation [35]. Martin et al. [35] found that characteristics of population dynamics such as population growth rates and peak densities were well captured by DEB theory. Their initial model, however, failed to reproduce the decline in population size that follows population peak density. The decline phase was addressed by further assumptions of stage dependent mortality related to resource availability. Our model exceeds the approach by Martin et al. [35] by an integration of the standard DEB model [34] with formulations of body-size scaling of starvation resistance [36]. In addition, for cross-level testing, each of the above hypotheses is represented by a sub-model in our IBM. At the level of individual organisms, we tested how well the different sub-models describe somatic growth and reproduction in *D. magna* under different conditions of resource availability and population density. At the population level, we evaluated how well laboratory population dynamics are predicted by the different sub-models that were parameterised using individual level data.

Systematically testing resource- and crowding-related hypotheses at the level of the individual organism and population level revealed that only operating different hypotheses together enabled accurate model predictions of *D. magna* population dynamics and size structure. Our study provides an example of how different hypotheses can be formulated based on first principle rules of metabolic organisation and how individual-based population models can facilitate hypothesis testing across different levels of biological organisation.

## Materials and Methods

### Model Description

**Figure 1:**
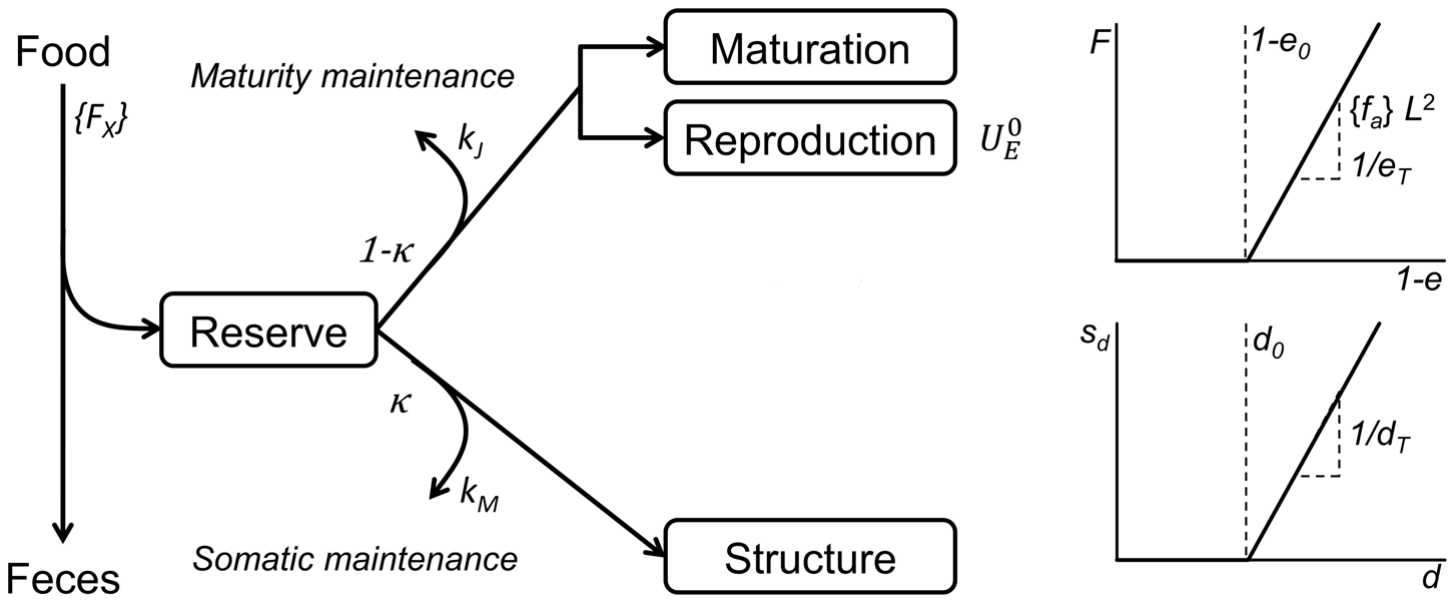
Schematic representation of the DEB model. Given parameters are involved in different resource-related and crowding-related hypotheses respectively (see Table 1). For modelling adaption of food ingestion and change in energy allocation, we applied an adaptive plasticity function F (upper right panel) to the respective parameter, where the level of plasticity increases with decreasing scaled reserve density *e* (or rather increasing 1-*e*) beyond a threshold (dashed line). Similarly, we used a stress function s (lower right panel) for modelling crowding effects at increasing population density *d*. More detailed explanations are given in the main text.

For hypothesis testing, we used an IBM, where *Daphnia magna* individuals are represented based on DEB theory [12]. At the individual level, our model quantitatively describes rates at which energy is assimilated from food and allocated via a reserve compartment to maintenance, structure and the reproductive system (Figure 1). During the course of time, if supplied with sufficient food, an individual increases in structure, i.e. grows, matures and when it reaches puberty, maturation stops and reproduction starts. At the population level, modelled individuals compete for food and space, whereby similar to real life, population dynamics emerge from individual-level properties and interactions. A detailed description of the model, the underlying variables as well as the source code (implemented in Delphi XE2, Embarcadero Technologies San Francisco, USA, 2011), is provided in the Supporting Information S1. An extensive derivation of DEB theory has been published by Jager [37], whereas discussions on underlying concepts can be found in [38], [39].

In accordance with the DEB concept of physiological modes of action [40], we considered several options for the effects of resource-related adaptive plasticity and crowding on the acquisition and use of energy from food. Each of our hypotheses is represented by a sub-model within the IBM which changes a model parameter proportional to a certain level of resource-related or crowding-related stress; for an overview see Table 1.

**Table 1:** Hypotheses for resource-related adaptive plasticity and crowding-related mechanisms.

No.	Description	Parameters	Equations
H_0_	No resource-related plasticity, no crowding-related stress	–	–
*Resource-related hypotheses*			
H_1_	Filtration rate increased	{*F_X_* }	eq. 1, eq. 2
H_2_	Energy allocation changed	*κ*	eq. 1, eq. 3
H_3_	Filtration rate increased and energy allocation changed	{*F_X_* }, *κ*	eq. 1, eq. 2 & eq. 3
*Crowding-related hypotheses*			
H_4_	Filtration rate decreased	{*F_X_* }	eq. 4, eq. 5
H_5_	Maintenance costs increased	*k_M_, k_J_*	eq. 4, eq. 6
H_6_	Filtration rate decreased and cost for reproduction increased	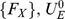	eq. 4, eq. 5 & eq. 7

Model parameters that are assumed to be affected and equations that are involved are additionally listed. See Table 3 for parameter descriptions.

For plasticity sub-models, we assumed that *Daphnia* individuals might adapt to conditions of low food by either increasing their filtration rate or by changing the rule for energy allocation within the body, or via both increased filtration and changed energy allocation. We moreover assumed that adaptive processes depend on the amount of reserve stored in the organism and relate the filtration rate and the amount of energy allocated to soma, to the scaled reserve density, *e* (which can have values between 0 and 1). Therefore, plasticity starts when the scaled reserve density drops below a threshold density. To obtain a positive relationship between plasticity and reserve, we used the “one minus” scaled reserve density (1-*e*) and accordingly, a threshold for plasticity of 1-*e_0_* in the formulation of the threshold function *z_e_*:
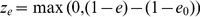
(1)


The threshold function, multiplied by a factor for adaption, indicates the level of plasticity *F* on a model parameter as described below (see also Figure 1).

In our first hypothesis (H_1_), we assume that daphnids adjust their filtration rate in response to resource shortage. In *Daphnia*, mechanical sieving is the dominant process of food intake, with filtering limbs retaining suspended food particles that are larger than the mesh size of the filters [41]. In the presence of food, increasing the filtration rate leads to an increase of the feeding (ingestion) rate. Two previously observed processes thus formed the basis for the formulation of hypothesis H_1_∶1) starved daphnids were shown to almost immediately adjust their filtration rate after addition of food by increasing their appendage beat rate [27], and 2) daphnids are able to increase ingestion by adapting the size of filter-screen areas to changes in food availability with a lag-time equal to inter-moulting periods [28]. As an approximation, we combined these two processes and assumed that the filtration rate is updated on a daily basis, depending on the amount of reserve stored in the body. Therefore, the scaled filtration rate {*F_x_*} was assumed to increase linearly with decreasing scaled reserve density, *e*, below the threshold, or rather increase with increasing 1-*e* beyond the threshold of 1-*e_0_* (see eq. 1). The increase in filtration rate then depends on a factor for adaptive plasticity {*f_a_*} and the animal structural surface area L^2^, which is a reasonable assumption as the size of filter-screens was found to depend on body size [11]. The actual scaled filtration rate is then given by equation 2:
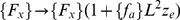
(2)


In hypothesis H_2_, we follow the observation of Guisande and Gliwicz [26] that somatic growth increased while reproductive output decreased, together with decreasing environmental food concentrations. Within the DEB scheme, partitioning of energy follows the κ-rule, assuming that a portion κ of the mobilized reserve is used for somatic maintenance and growth, while the remaining fraction 1- κ serves for maturity maintenance, maturation and reproduction. A switch in the energy allocation toward soma (increasing κ) can explain the above life history shift, because increasing κ allows for increased growth, and at the same time the fraction of reserve mobilized for maturation and reproduction is reduced. In H_2_, we therefore assumed that the fraction of energy allocated to soma, κ, increases if food is scarce or absent (and as a consequence 1- κ decreases). Similar to the filtration rate formulation above, we assumed that κ changes with the one-minus-the-scaled-reserve density (1-*e*) beyond the threshold for plasticity (1-*e_0_*), proportional to the tolerance scaled reserve density *e_T_*. The value of κ is limited to a maximum of 1 (eq. 2):
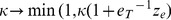
(3)


In our formulation of hypotheses H_3_, both adjustment of filtration rate (eq. 1) and changed energy allocation (eq. 2) act simultaneously.

For modelling crowding-related fitness, we introduced a stress function *s_d_* that indicates the level of stress on a model parameter caused by population density *d_P_* (eq. 3).
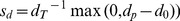
(4)


The stress level, therefore, increases in a linear way beyond the no-effect population density *d_0_*_,_ proportional to the tolerance density, *d_T_*. This is a modification of the stress function used in ecotoxicology, where the stress level increases in proportion to the toxicant concentration [42]. The stress function was subsequently applied to the different parameters in accordance with the respective hypotheses. As a consequence, we assumed that all individuals in a given population are equally affected by crowding (in contrast, the plasticity hypotheses are related to the reserve status of the individual, which can vary among individuals). In hypothesis H_4_, we assumed that the filtration rate decreases at high densities (eq. 4), whereas in hypotheses H_5_, high densities were assumed to increase costs for somatic maintenance *k_M_* and maturity maintenance *k_J_* (eq. 5). In H_6_ (eq. 4 and eq. 6), we hypothesised that filtration rate is decreased and that the production costs of a single egg 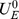 is increased (i.e. reflecting that daphnids might produce larger eggs at high population densities [32]). Since an individual only has a certain amount of energy available in its reproduction buffer, increased costs for single eggs will result in smaller clutch sizes.
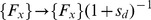
(5)




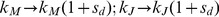
(6)




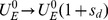
(7)


DEB models describe an individual based on state variables of structure, reserve and maturity. Structure and reserve both contribute to biomass (whereas maturity specifies the developmental status only), whereby only structure requires maintenance and only reserve fuel metabolic processes [43]. Because of their rather abstract nature, none of these state variables can be measured directly, however, they link to observable traits such as body size or time to first reproduction. Accordingly, most model parameters are not directly observable, but can be estimated from empirical response variables such as feeding rate, assimilation rate, growth, reproductive output and survival (for a discussion, see [37]). For parameter estimation, we first fitted feeding and assimilation sub-models (see Supporting Information S1) to experimental data for filtration rate, functional response and assimilation rate. With fixed parameters for feeding and assimilation, we simultaneously estimated parameters for reserve dynamics, growth, reproduction (see Supporting Information S1) and adaptive plasticity (eq. 1, eq. 2) using data for size-at-age and cumulative reproduction for different food levels. Thereafter, we derived ageing and survival parameters (see Supporting Information S1) based on data for starvation and survival under high food conditions. Finally, we estimated crowding parameters (eq. 3) used in the different hypotheses (eq. 4– eq. 6) from observations of size-at-age and cumulative reproduction for daphnids that were kept at different population densities and supplied with (close to) *ad libitum* food. As mortality decreased the population density during the course of the original experiment, we used survival data as input variables for the model fit.

We applied the simplex method as developed by Nelder and Mead [44], using weighted least squares [38] as criteria for estimating parameters related to energy uptake, reserve dynamics, growth and reproduction, and maximum likelihood [45] for the estimation of survival parameters. For the evaluation of model performance, we used the Nash-Sutcliffe model efficiency, NSE [46] as an indicator for the goodness-of-fit of individual-level submodels and as a quality measure for the accuracy of model predictions at the population level. The NSE can reach a maximum value of 1 (perfect fit or prediction) and also exhibit negative values, with NSE = 0, meaning that the model is performing as well as the arithmetic mean of the data.

### Laboratory Experiment

Most individual-level data used for model parameterisation were derived from the literature (see Figure captions). However, in this study, we performed a single experiment for the parameterisation of the starvation submodel and to estimate the dry-weight-to-length relationship. *Daphnia magna* individuals were cultured as described by Siehoff et al. [47]. For the starvation experiment, 10 medium- (two-week-old) and 10 large- (five-week-old) sized daphnids were randomly selected from the culture and individually kept without any food in test vessels containing 20 mL M4 media [51]. Body size, excluding the spine, was measured under magnification at the start of the experiment. Survival was checked daily and the dry weight (75°C, 24 h) of dead animals was determined. To obtain a length-to-weight relationship for well-fed animals, we additionally measured body length and dry weight of 70 daphnids that were sampled from the culture.

## Results

**Figure 2:**
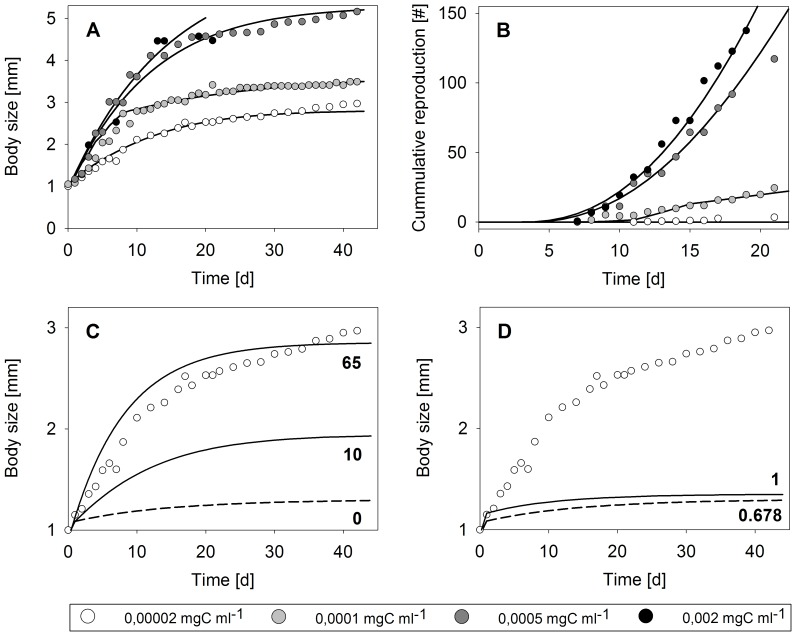
Somatic growth and reproduction under different food conditions (mg carbon) in flow-through systems. Somatic growth was measured as increase in body length (excluding spine) during the course of time whereas cumulative reproduction is given as mean offspring number produced per daphnid during a certain period of time (data from [48]). A and B: total data, model fit based on hypothesis H_3_; C: low food for different values of filtration adaptive plasticity factor ({*f_a_*}, hypothesis H_1_); D: low food for different values for κ (hypothesis H_2_); solid lines represent modelled growth and reproduction considering resource-related adaptive plasticity; dashed lines in C and D represent model without plasticity (hypothesis H_0_).

**Figure 3:**
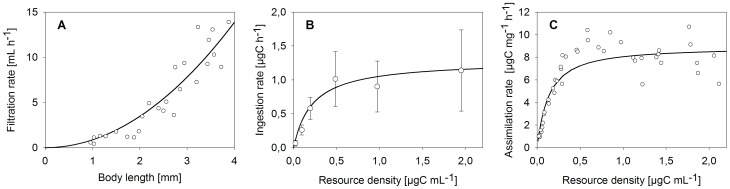
Feeding and assimilation. A: Filtration rate as a function of daphnid body length, measurements excluding spine, B: ingestion rate as function of algal resource density and c) assimilation rate per unit of *Daphnia* dry weight as function of resource density; data in A and B from [48], data in C extracted from [49].

The DEB model predicts that growth and maximum size as well as reproduction increase with increased resource availability and food assimilation. A comparison of the model fits with experimental data for somatic growth (size-at-age data) and cumulative reproduction, i.e. the mean number of offspring produced per female during a certain period of time, is provided in Figure 2. Model fits and data for filtration rate as function of body size as well as ingestion and assimilation of carbon as function of resource availability (carbon density) are shown in Figure 3. The original experiments for *D. magna* feeding, growth and reproduction were conducted in 500 mL test vessels at flow-through conditions using a flow rate of 360 mL h^−1^ [48]. Assimilation rates as function of resource density were originally determined in batch experiments by Bohrer and Lampert [49]. With fixed parameterisation for feeding and assimilation sub-models (Figure 3), and without any additional assumption of resource-related plasticity (hypothesis H_0_), our model is able to represent size-at-age and cumulative reproduction data for different food levels fairly well (Figure 2, Table 2). However, when ignoring adaptive plasticity, growth is underestimated for the lowest level of food availability (Figure 2 c, d). Assuming a higher filtration rate (hypothesis H_1_) can account for this difference between the observed growth and the model fit based on the null hypothesis. Therefore, an increase in the related parameter, i.e. the filtration rate adaptive plasticity factor, can lead to an increase in maximum size (Figure 2c). A value for the filtration rate adaptive plasticity factor of {*f_a_*} = 10 means, for instance, that a daphnid with a body length of 1.7 mm and a scaled reserve density of e = 0.5 (corresponding to an assimilation rate of 0.006 mgC d^−1^ if food availability is constant) would be able to double its ingestion rate. A factor of {*f_a_*} = 65 described size-at-age data for low food conditions well (Table 2), although it overestimated initial growth (Figure 2 c). Another option of adaptive plasticity might be that daphnids increase the fraction of energy allocated to soma, κ under low food conditions (hypothesis H_2_). However, increasing κ to a maximum possible value of 1 (meaning that all energy is allocated to growth and somatic maintenance) only marginally increased growth at low food availability and did not provide an adequate model fit (Figure 2 d). Simultaneously assuming an increased filtration rate and altered energy allocation (hypothesis H_3_) led to an increased resource assimilation and subsequently to a larger amount of reserve that can be allocated to somatic maintenance and growth (although the allocated fraction is the same). Increasing filtration by a factor of {*f_a_*} = 10.4 (≈ doubling the ingestion rate for the case of a 1.7 mm daphnid, see above) and increasing the fraction of energy allocated to soma (κ), compared to the ‘baseline’ κ = 0.678, resulted in a high model efficiency for adaptive plasticity (Table 2) and overall reflected the size-at-age and cumulative reproduction data well (Figure 2 a, b). Parameter descriptions and values for the model fit according to the H_3_ hypothesis are given in Table 3.

**Table 2:** Performance of different hypotheses.

	Individual-level data		Population-level data	
No.	NSE growth	NSE reproduction	NSE high food	NSE low food
H_0_	0.691	0.957	−0.063	0.974
H_1_	0.968*	0.980*	−14.796	−0.216
H_2_	0.693	0.949	−0.295	0.975
H_3_	0.975	0.983	0.307	0.937
H_4_	0.974	0.861	−2.606	0.791
H_5_	0.995	0.925	0.420	0.989
H_6_	0.995	0.933	0.621	0.985
H_3_+H_4_	n.a.	n.a.	0.207	0.908
H_3_+H_5_	n.a.	n.a.	0.736	0.987
H_3_+H_6_	n.a.	n.a.	0.832	0.970

Nash-Sutcliffe model efficiency (NSE) of sub-models representing different hypotheses (H_0_–H_6_, see Table 1) for individual-level data and total population abundance at high and low resource availability, respectively.

*assuming a filtration adaptive plasticity factor of {*f_a_*} = 65 (see Figure 2c); n.a.: not applicable.

**Table 3:** Parameter estimates and confidence intervals (CI) for the individual-based model.

Symbol	Description	Value	95% CI	Unit
*α*	Median of threshold distribution	0.41	0–2**	[−]
*β*	Slope of threshold distribution	4	0–10**	[d^−1^]
δ_M_	Shape coefficient	0.54*	n.e.	[−]
*d_Vd_*	Density of dry weight	0.014	0–0.048	[mg mm^−3^]
[*E_G_*]	Volume-specific cost for structure	0.00179	0.00163–0.00195	[mg mm^−3^]
*e_0_*	Scaled reserved density threshold for plasticity	0.63	0.55–0.67	[−]
*e_T_*	Tolerance scaled reserve density for plasticity	0.139	0.0001**–0.873	[−]
{*F_x_*}	Surface-area-specific filtration rate	2.97	2.85–3.10	[ml mm^−2 ^h^−1^]
{*f_a_*}	Surface-area-specific filtration rate adaptive plasticity	10.4	6.3–15.8	[mm^−2^]
*h_a_*	Ageing acceleration constant	0.00029	0.00008–0.00034	[d^−2^]
*ill*	Incipient limiting level	0.00036	0–0.342	[mg ml^−1^]
*κ*	Allocation fraction to soma	0.678	0.612–0.769	[–]
*k_d_*	Damage recovery rate constant	0.09	0–0.834	[d^−1^]
*k_J_*	Maturity maintenance rate coefficient	0.969	0.913–1.051	[d^−1^]
*k_M_*	Somatic maintenance rate coefficient	1.599	1.453–1.739	[d^−1^]
*k_R_*	Reproduction efficiency	0.95*	n.e.	[–]
*L_i_*	Initial volumetric structural length	0.0000001	n.e.	[mm]
*ϖ_d_*	Contribution of reserve to body weight	1.6	0–7.8	[–]
{*p_Am_*}	Surface-area-specific maximum assimilation rate	0.0145	0.0117–0.0164	[mg mm^−2 ^d^−1^]
*p_Xmin_*	Minimum assimilation efficiency	0.54	0.27–0.92	[−]
*p_Xmax_*	Maximum assimilation efficiency	0.95	0.71–1.00	[−]
*s_G_*	Ageing stress coefficient	0.40	0.31–0.51	[−]
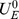	Cost of an egg	0.089	0.0826–0.0964	[mm^2^ d]
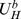	Scaled maturity at birth	0.012	0.007–0.017	[mm^2^ d]
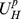	Scaled maturity at puberty	0.20	0.17–0.23	[mm^2^ d]
*v*	Energy conductance	0.825	0.770–0.874	[mm d^−1^]
*X_K_*	Half saturation constant	0.00022	0–0.001**	[mg ml^−1^]

Parameter estimation was based on the submodel representing the combined adaptive plasticity hypothesis (H_3_).

*fixed according to [12]; **limit set in parameter estimation; n.e.: not estimated.

**Figure 4:**
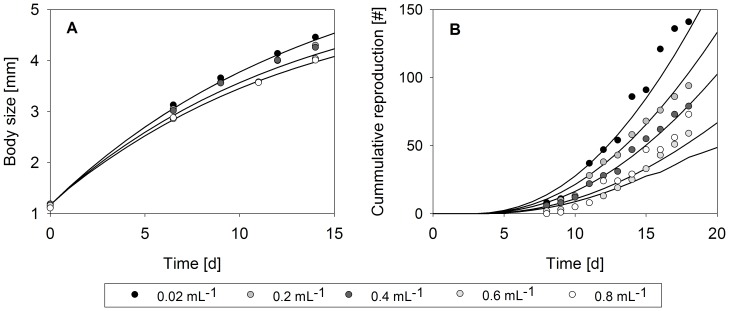
Somatic growth in terms of body size and cumulative reproduction (mean offspring number per female) for different crowding conditions (daphnids per mL). Model fit (lines) is based on the assumption of reduced filtration rate and increased costs for reproduction (hypothesis H_6_); data (circles) from [31]; note that in B the data for highest density (white circles) relate to lowest predicted cumulative reproduction; high density reproduction data (at 0.8 mL^−1^) were not included in model parameterisation.

Model fit (for hypotheses H_6_) and experimental data [31] for growth and cumulative reproduction at different crowding conditions are shown in Figure 4. To obtain different crowding conditions, in the original study, daphnids were kept in 50 mL test vessels individually or in groups of up to 6 individuals at flow-through conditions using high food densities to avoid resource limitation (algae density: 0.001 mgC mL^−1^; flow rate: 30 mL h^−1^) [31]. All of the sub-models representing crowding-related hypotheses (H_4_, H_5_ and H_6_) predict that individual fitness decreases with increasing population density and thereby describe size-at-age and cumulative reproduction data fairly well (Table 2). However, assuming a decline in filtration rate and increased reproductive costs (hypothesis H_6_) provided the best representation of the data (Figure 4). Parameter estimates for the threshold population density *d_0_*, which marks the onset of a crowding-related effect, indicated that in hypothesis H_6_, the filtration rate might be disrupted at high population densities (≥370 daphnids L^−1^), whereas costs for reproduction increase from comparably low densities (≥21daphnids L^−1^). Parameter values for the different crowding-related sub-models are provided in Table 4.

**Table 4:** Parameter estimation for crowding mechanisms.

No.	Parameter affected	*d_0_*	*d_T_*
H_4_	{*f_x_*}	0.02	0.106
H_5_	k_J_	0.021	0.29
H_5_	k_M_	0.15	2.05
H_6_	{*f_x_*}	0.37	0.95
H_6_	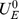	0.021	0.59

Two parameters, the no-effect population density *d_0_* [# ml^−1^] and tolerance population density *d_T_* [# ml^−1^], were used in submodels representing different crowding-related hypothesis (H_4_–H_6_).

Variable resource availability e.g. as a consequence of competition and a reduction in food ingestion or increase in maintenance costs due to crowding has consequences for individual survival. Under the assumption that *D. magna* individuals solely fulfil maintenance requirements and cease energy allocation to maturation and reproduction (H_2_ and H_3_) when starved, the model predicts that large individuals resist starvation longer than smaller conspecifics. Survival data and corresponding model fits are shown in Figure 4. The survival sub-model fits empirical data for both starvation and high food conditions well (Figure 4 a, b). In addition, weight loss during the time course of starvation is well described by the model (Figure 4 c).

**Figure 5:**
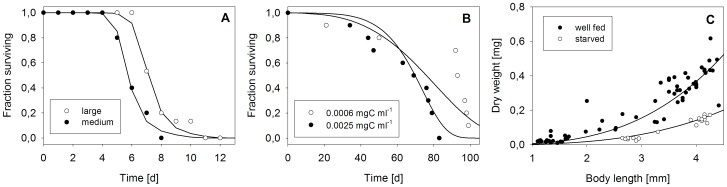
Survival and *D. magna* body sizes based on hypothesis H_3_. A: Fraction of survivors as function of time of medium- (2.9±0.2 mm) and large- (4.1±0.1 mm) bodied daphnids in the absence of food, B: survivorship for daphnids that were supplied with two different (high) amounts of algal food, C: dry weight as function of physical body length for daphnids that were kept under culture conditions (black dots) or that were starved until death (white dots); dots and lines represent empirical data and model fit respectively; data in A and C this study and data in B published in [14].

**Figure 6:**
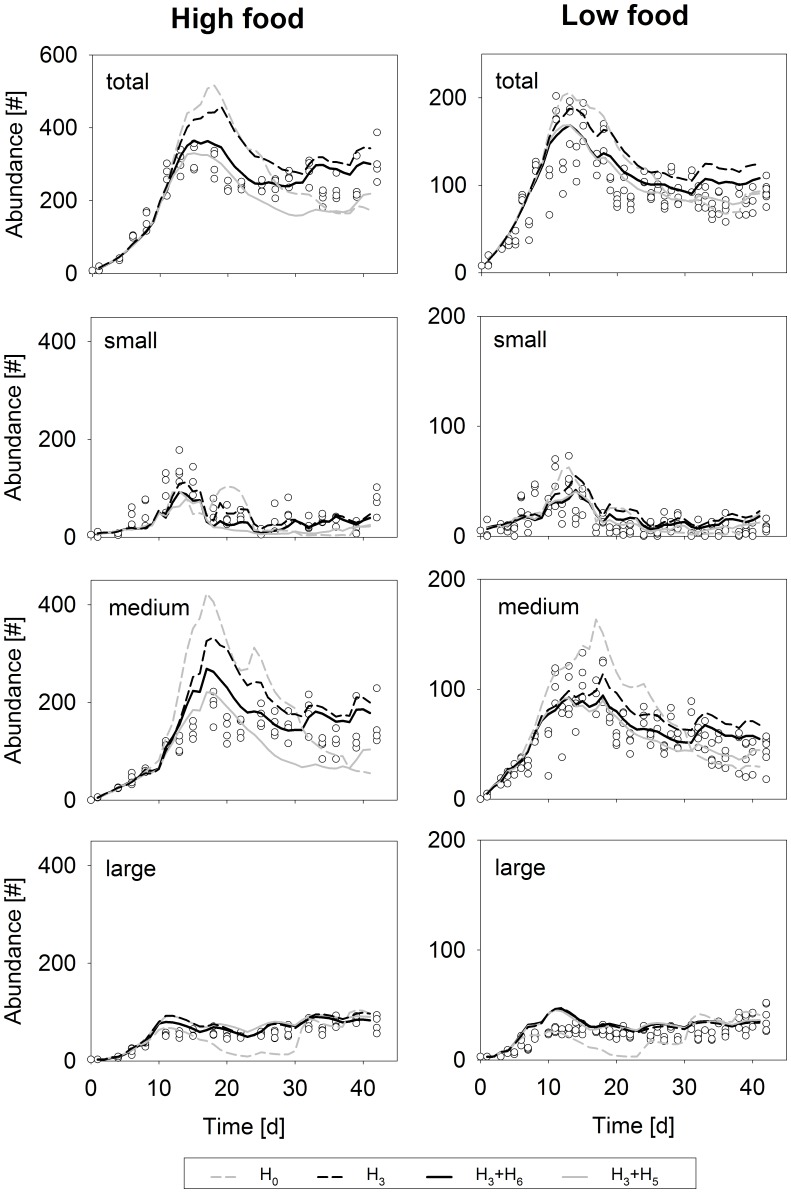
Population level test of hypotheses. Population-level consequences of different hypotheses (see Table 1) are shown for high (1.3 mgC per day and population) and low food (0.5 mgC per day and population) conditions respectively; empirical data (dots) and model predictions (lines) are given for total abundances (population size in total numbers) and abundances (numbers) within three size classes: small <1.25 mm; medium 1.25–2.10 mm, large ≥2.10 mm; for reasons of clarity, not all hypotheses and combinations are shown; but see summary statistics in Table 2; data from [50].

We compared model predictions for different hypotheses with independent data [50] of laboratory-scale population dynamics for low and high food conditions (Figure 5). In the original study, population dynamics were assessed by counting total numbers and numbers for different *D. magna* size classes (see caption Figure 6) during a period of 42 days. Population experiments were carried out in 900 mL test vessels, starting with five newborn daphnids (<24h) and three adults, and food (for densities see caption Figure 6) being added daily on work days and tripled on Fridays [50]. The chosen laboratory data set allows the evaluation of three key features of population dynamics, i.e. initial population growth followed by a peak density and after a phase of down-regulation, by an equilibrium population density. The individual-based model closely predicted initial population growth and initial population size distribution irrespective of the hypothesis applied. For low food conditions, all of the sub-models also make reasonable predictions for peak density and equilibrium population size (Figure 4, Table 2), with the exception of hypothesis H_1_, where equilibrium density is overestimated by a factor of about three. In contrast, for conditions of high resource availability, leading to higher population densities, none of the single hypotheses representing sub-models is able to predict population total abundance well (Table 2). The null hypothesis overestimates population peak density and underestimates equilibrium population size. Applying the combined adaptive plasticity hypothesis (H_3_) buffers the down-regulation that follows the peak density to some extent compared to H_0_, but generally overestimates population density. Only combinations of resource-related and crowding-related hypotheses (H_3_+H_5_ and H_3_+H_6_) give reasonable predictions of total population abundances (Table 2) and population size structure (see Figure 5), while simultaneously representing observed individual growth at low food availability. However, applying H_3_+H_5_ sub-models underestimates total population abundance at equilibrium, as indicated by population size structure, due to lower reproduction and lower subsequent survival of intermediate-sized daphnids predicted by this model (Figure 5).

## Discussion

Overall, none of our initial six hypotheses concerning how resource-related adaptive plasticity and crowding-related fitness might cause individual life history variability could alone predict *Daphnia magna* population dynamics well. Instead, a combination of plasticity mechanisms and crowding effects is suggested to play a role in individual life history variation and the regulation of dense populations, which in particular, might occur in nutrient-rich environments. Our results also indicate that adaptive plasticity and crowding play only a minor role in the regulation of the population in conditions of low resource availability.

A general framework for testing how environmental variability affects individual life history is provided by DEB theory (examples are given in [12]). In the DEB scheme, stress is regarded as a change in one or more energetic parameters that are related to processes involved in the chain of energy acquisition from the environment and its subsequent distribution and use in the organism. These processes include finding and ingesting food, assimilating and incorporating energy into the reserve and subsequently using the reserve for growth, reproduction and maintenance (Figure 1). This concept of physiological modes of action [40], which was originally developed for toxicity assessment, is basically applicable to different types of stresses that are quantifiable as concentration or density. The concept provides a suite of models that allow an analysis of the consequences of alterations in energy uptake and allocation patterns in response to a stressor. Since animals are regularly exposed to multiple compounds and interactions, it is plausible to assume that a stressor exerts alterations in energy allocation only when the stress level exceeds a certain threshold [37]. Beyond the threshold, there will be a change in one or more model parameters corresponding to the respective physiological mode of action. In our resource-related and crowding-related hypotheses, we considered four different modes: changed feeding, changed energy allocation, increased costs for somatic and maturity maintenance and increased costs for reproduction. As predicted by the theory, decreased feeding and increased costs for somatic and maturity maintenance will result in decreased growth and reproduction as well as in prolonged juvenile development; in turn, growth and reproduction can increase with increasing feeding rates. In contrast, increasing the fraction of reserve allocated to soma leads to increased growth, while at the same time decreasing the reproductive output and prolonging juvenile stage duration. Lastly, increasing the costs for reproduction decreases the offspring number, whereas growth and juvenile development remain unaffected. In the following, we will make use of these predictions to discuss individual-level data and cross-level consequences of resource-related adaptive plasticity and crowding-related fitness.

### Test of Resource-related and Crowding-related Hypotheses at the Individual Level

For adaptive plasticity, we tested whether increased feeding activity and altered reserve allocation either alone or together (hypotheses H_1_–H_3_) can account for the difference between the observed growth at low food availability and the model fit based on the null hypothesis. Strictly speaking, the concept of physiological modes of actions is not applicable to resource-related adaptive plasticity because there is a negative relationship between food density (or rather the lack thereof) and the physiological or morphological response. We overcame this problem by linking changes in parameter values to the lack of reserves in the organism; the conceptual consequences for energetics are then identical to effects caused by internal toxicant concentrations, for which the concept was originally developed [40]. In filter feeders such as *Daphnia*, the rate of food ingestion depends on a critical environmental food concentration, the incipient limiting concentration [52]. Above the incipient limiting concentration, food intake is constant e.g. due to morphological and digestive constraints, whereas an ingestion rate below the incipient limiting concentration is limited by the maximum amount of water an individual can filter [53]. With immediate behavioural changes on one hand, daphnids can adapt the beat rate of filtering limbs in response to variable environments, such that the filtration rate decreases during starvation and increases after food addition [27]. With morphological changes on the other hand, the size of filtering limbs can be adapted to conditions of low food supply, a mechanism that is well known for a range of *Daphnia* species [11], [54]. Daphnids can change the size of filtering limbs when moulting, by changing the length of the seta in response to food concentration [28]. In contrast to behavioural changes, this long-lasting morphological alteration might explain the increase in individual growth at low food conditions that becomes apparent from the mismatch between growth data and our model prediction based on the no-plasticity hypothesis H_0_ (Figure 2). For instance, *Daphnia pulicaria* individuals that were kept at low food conditions increased their filter screen area by 41% and thereby almost doubled their ingestion rate, compared to that in high food conditions [55]. Given the relatively low ability of *D. magna* to increase filtering limb size compared to other species [11], assuming a filtration rate adaptive plasticity factor of ∼10 in our model (approximately doubling the filtration rate, see results) is quite optimistic for this species. As only a filtration rate adaptive plasticity factor of 65 provides a reasonable model fit (Figure 2c), hypothesis H_1_ does not fully account for increased growth at low food conditions. Some evidence shows that *Daphnia* individuals can adapt their energy allocation strategy to conditions of low resource availability [26], a hypothesis that we addressed by applying our H_2_-submodel. Evidence for a change in energy allocation is provided by a study of Bradley et al. [56], where the authors found that in *D. magna*, growth continues but reproduction ceases upon starvation. However, assuming that 100% of the reserve can be allocated to soma does not explain the increased growth that was observed under low food conditions, because the overall amount of energy assimilated by the individual is too low in this model. With increased food ingestion as a result of increased filtration rate due to morphological changes, a daphnid has more overall energy available, which can be allocated within the body. Assuming increased feeding together with an increased fraction of reserve allocated to soma (hypothesis H_3_) can therefore account for the observed growth patterns under all tested food conditions (Figure 2a). Increasing the fraction allocated to the soma at the same time, means that there is a lower fraction of reserve available for allocation to maturation and reproduction. The reproduction data reveal that the allocation of energy to the reproductive system might not completely cease in the low food conditions used in the experiment, as at least some offspring were produced. As the value of κ reached 1 at this food level, we did not account for this observation in our model.

Crowding effects can be related to population density or to the concentrations of chemicals released by individuals (if they are known). A number of crowding-related life history alterations have been reported, including reduced growth and reproduction as well as increased mortality [17], [18], [29], [30], [31]. In our crowding-related hypotheses, we tested whether these alterations might be a consequence of feeding interference (H_4_), increased maintenance costs (H_5_) or a combination of decreased feeding and increased costs for reproduction (H_6_). A reduction in food ingestion as well as an increase in maintenance costs in principle can account for the reduced growth and reproduction that has been observed for increasing *D. magna* population densities (Figure 3). According to DEB theory, decreased feeding and increased maintenance costs are also associated with delays in maturation, which become apparent as a longer time to first reproduction and were observed for densities above 400 individuals L^−1^ [18]. Accordingly, Goser [31] did not observe a disruption of feeding rates at lower *D. magna* densities. However, decreased feeding rates in crowding conditions have been reported for several *Daphnia* species [57], [58], [59]. Assuming that the feeding rate in *Daphnia magna* is reduced when population densities exceed 370 individuals L^−1^, our fitted model can account for the pattern observed for the crowding-related reduction in growth and the slight delay in maturation (Figure 3), but does not explain the full magnitude of the reproductive decline. In contrast, a crowding-related increase in the cost of reproduction would presumably lead to a lower number of produced offspring, whereas growth and maturation remain unaffected. Cleuvers et al. [32] found that *D. magna* individuals that were crowded produced larger eggs, resulting in a higher fitness of their offspring. A combination of reduced feeding and increased costs for reproduction that arise from producing larger eggs (H_6_) that are better equipped with reserve can explain the pattern observed for crowding effects on growth and reproduction. A consequence of this hypothesis is that due to crowding, the individual fitness of mother daphnids is reduced, whereas the fitness of their offspring might increase (for further discussions on this life-strategy shift by intra-specific interaction see [32]).

Resource-related adaptive plasticity and crowding-related fitness also have consequences for individual survival. If food ingestion decreases, e.g. due to resource depletion in the environment or due to crowding-related feeding interference, an animal is just able to survive at a certain threshold of ingested food and lower food levels will ultimately result in death by starvation. The DEB rules imply that the threshold food density depends on animal length. Therefore, small animals can outcompete larger conspecifics in constant environments, to an extent that is probably not realistic [60]. In natural zooplankton communities, large species usually dominate in the absence of predation [61]. Accordingly, positive relationships between body size and survivorship have frequently been reported to explain intraspecific variation in starvation resistance [36], [62], [63], [64]. To overcome this discrepancy, Kearney et al. [65] assumed that required food quality differs between conspecifics of different sizes in a way that favours older individuals. For example, Urabe and Sterner [66] highlighted the importance of food stoichiometry for *Daphnia* life history. Another solution to the problem is to consider survival to be stage-dependent [35], [67]. In our model, we link survival to reserve density via a physiological damage stage. With decreasing food ingestion, the damage increases and survival probability decreases. Upon starvation, energetics solely become a function of reserve dynamics. As in our parameterisation for *D. magna*, larger individuals exhibit slower reserve utilisation relative to smaller animals, and thus can resist starvation longer than their smaller conspecifics. If only considering food quantity, this can explain why a higher mortality in juveniles compared to adults is needed to adequately represent population demography in previous population model approaches [35]. In addition, adaptive plasticity can contribute to the competitive strength of larger daphnids in a twofold way. First, adapting filter-screens in relation to body size will enable larger individuals to be more efficient filterers than smaller ones. Second, the increased amount of assimilated energy subsequently allows a larger allocation of reserve towards the soma. In low food conditions, this results in more rapid growth, which can feed back to the individual’s efficiency as a filter feeder. In our parameterisation of the starvation model for *D. magna*, an individual is able to survive to some extent, although its reserve has been depleted. This basically implies that upon starvation, a daphnid reduces its energy expenditure or decomposes its structure. However, these processes are not considered in our model. It can be speculated that larger individuals can shrink to greater extents compared to smaller conspecifics, which might also contribute to the competitive strength of larger animals.

### Population Level Consequences of Adaptive Plasticity and Crowding Mechanisms

Resource-related life history variations are well documented in the scientific literature [12], [68], [69] and the question of how these feed back to population dynamics and the stability of consumer-resource interactions is a long-standing issue in ecology. For instance, combined developmental delay and mortality produce certain types of consumer-resource cycles. In theory, for population cycles with small amplitudes, slow juvenile development leads to a developmental delay that is typically longer than the cycle period; whereas for large-amplitude cycles, the cycle period exceeds the developmental delay [70]. For the prediction of population dynamics and consumer-resource interactions, it is thus important to know how responses to environmental changes affect traits such as juvenile stage duration and survival probability.

Within a population, competition for resources is strongest at the carrying capacity of a system, as in general, an increase in population density increases competition for resources [71]. Exceeding the carrying capacity results in starvation-induced mortality (of juveniles), followed by a decline in population size; the probability of death in a post-peak phase is thereby closely related to the oscillation amplitude [72]. The more the population size exceeds the carrying capacity, the larger the subsequent decline might be, which can result in large amplitude cycles for simple predator-prey systems, such as the daphnid-algae system [72]. Similarly, in our simulations for the no-adaption-no-crowding hypothesis (H_0_), exceeding carrying capacity to a great extent was followed by a rapid population decline for high food conditions, whereas the amplitude was smaller for low food simulations (Figure 4). As resource-related adaptive plasticity can extend starvation resistance especially in larger individuals, the mechanism of filtration adaption and altered reserve allocation buffered the population decline but not the peak population size, as revealed by our H_3_-simulation.

As well as resource-related adaptive plasticity, crowding-related mechanisms can buffer cycle amplitudes in nutrient-rich environments; however, they probably play a minor role in nutrient-poor environments. Assuming increased maintenance costs as a mechanism for crowding-related fitness implies that an increased fraction of reserve is spent on body functioning, whereas a lower fraction is available for growth, maturation and reproduction. Moreover, reserve depletion becomes faster upon starvation. When combined with adaptive plasticity submodels (H_3_+H_5_), increased maintenance costs therefore lead to low peak abundances and high mortality at carrying capacity, overall underestimating population equilibrium size in high food availability conditions. This suggests that either the slope of the response curves (represented by the parameter *d_T_* in the crowding stress function) must be lower, or that other mechanisms are more important.

An alternative model that also predicts population level data well is based on the hypothesis of feeding interference and increased cost for reproduction (H_6_). In this hypothesis, we considered higher costs of egg production to mimic larger eggs being produced under crowding conditions (see above). However, in the model, we ignored the fact that neonates hatching from these eggs are larger and probably better equipped with reserves than individuals produced at lower population densities. Moreover, we largely ignored other factors that might influence egg size. For instance, egg size was shown to increase with maternal size [73] and smaller eggs at high food availability were also observed, but interclonal differences exist [5]. In the initial phase of the population experiments (in Figure 6), daphnids are relatively small and food is abundant. As smaller offspring size at abundant food is the opposite of the DEB prediction, this can explain why we initially underestimated reproduction and subsequently overestimated the peak abundance of medium-sized daphnids (Figure 6). The neonates initially produced in our models are probably larger and therefore fitter, than those produced in real life.

The individual-level model fits for H_5_ and H_6_ suggest that mechanisms that underlie the decline in reproduction and growth might operate at different population densities (as indicated by the parameter *d_0_* presented in Table 4). For hypothesis H_6_, this implies that costs for reproduction are increased at relatively low population densities and can therefore play a role in the population regulation in rich as well as in poor environments. In contrast, in the H_3_+H_6_-submodel, feeding interference is ‘switched on’ at the very peak density of high food simulations, and thus might solely play a role in nutrient-rich environments.

*D. magna* is well known as a cyclic parthenogenic species. In particular, crowding, photoperiod and to some extend food shortage were reported to induce (seasonal) production of males [74], [75]. In the current modeling approach we only considered asexual reproduction of female daphnids as males were hardly produced in the laboratory settings used in the original studies our model was compared to (see e.g. [32]). However, sexual reproduction as well as the production of resting eggs need to be incorporated when e.g. modelling population dynamics under more realistic conditions or exploring the consequence of sexual reproduction, such as recombination, on individual characteristics across several generations.

### Implications of the Multiple Hypothesis Approach

This study provides an example of how individual-based population models that are based on first principle rules of metabolic organisation might be used in hypothesis testing across different levels of biological organisation. We made use of the concept of multiple working hypotheses [76], with the aim of identifying models that offer plausible approximations of empirical observations. In general, the method is based on the idea that empirical data support one or more *a priori*-defined hypotheses, while providing less evidence for others. In this way, repetitions of the data-gathering and multiple hypotheses-testing process will finally lead to advances in science [77]. We have formulated hypotheses and combinations of hypotheses (models) of different complexity, including a null hypothesis (which is classically not involved in the concept of multiple working hypotheses). The aim was to test the contribution of certain alternative hypotheses to the empirically observed patterns across biological levels of organisation. It can be argued that our process of comparing different hypotheses might be biased by model complexity or the number of parameters involved in the different submodels, including the problem of under- or overfitting models (for a discussion on the model selection problem, see [78]). However, our aim was predominantly to test the significance of certain individual-level processes, as discussed in the scientific literature, rather than finding the model that best fits the empirical data. Several methods are available for hypotheses or model selection, including the cross-validation method (for an overview, see [79]). The basic principle of cross-validation is to divide empirical data into two subsets, one of which is used for model parameterisation and the other is used for model testing. As individual-based models simulate population dynamics based on individual properties, they offer a natural way of data division for cross-validation: individual-level observations can be used for parameterisation, while the emergent model output can be tested against independent population-level data.

Possible mechanisms underlying population dynamics and not least density dependence, which is a necessary element for the formation of cyclic population fluctuations, have been well described in theoretical terms in text-books [80], [81]. As exemplified above, various proposed mechanisms of density dependence have been explored independently of each other in controlled laboratory experiments. However, designing experimental approaches to actually explore and test different hypothetical mechanisms of density dependence against each other is not straightforward (for a discussion on experimental designs, see [71]), which is reflected by a limited number of empirical studies in the literature that address this issue (but see [82]). It has been previously suggested to use mechanistic population models to test possible factors that might drive cyclic population dynamics, against field or laboratory data on population fluctuations [83]. However, to our knowledge, the present study is the first attempt to concurrently test several hypotheses on density-dependence mechanisms using different organisational levels for the evaluation of model performance to suggest the most probable mechanism(s). As the modelled population dynamics emerge from the individual-level life-history traits, energy acquisition, energy allocation, and to some degree behaviour, this type of model can be used to generate general insights into the regulation of population dynamics under specific environmental conditions. Such understanding is crucial for the assessment of manmade stressors in the environment, as well as for the management and eventual conservation of populations and thereby biodiversity in ecosystems.

## Supporting Information

Supporting Information S1 **Model description and source code.**
(PDF)

## References

[1] Schoener TW (2011) The newest synthesis: understanding the interplay of evolutionary and ecological dynamics. Science 331: 426–429. 21273479 10.1126/science.1193954

[2] Stelzer CP (2012) Population regulation in sexual and asexual rotifers: an eco-evolutionary feedback to population size? Funct Ecol 26: 180–188. 10.1111/j.1365-2435.2011.01918.x PMC7611971 34764531

[3] Miller RS (1967) Pattern and process in competition. Adv Ecol Res 4: 1–74.

[4] McCauley E, Murdoch WM (1987) Cyclic and stable populations: plankton as paradigm. Am Nat 129: 97–121.

[5] McCauley E, Nisbet RM, Murdoch WW, De Roos AM, Gurney WSC (1999) Large-amplitude cycles of *Daphnia* and its algal prey in enriched environments. Nature 402: 653–656.

[6] Stearns SC (1989) Trade-Offs in Life-History Evolution. Funct Ecol 3: 259–268.

[7] Roff DA, Fairbairn DA (2007) Laboratory Evolution of the Migratory Polymorphism in the Sand Cricket: Combining Physiology with Quantitative Genetics. Physiol Biochem Zool 80: 358–369. 17508332 10.1086/518012

[8] Glazier DS (1992) Effects of food, genotype, and maternal size and age on offspring investment in *Daphnia Magna* . Ecology 73(3): 910–926.

[9] Marshall DJ, Keough MJ (2009) Does interspecific competition affect offspring provisioning? Ecology 90(2): 487–495. 19323232 10.1890/08-0320.1

[10] Allen RM, Buckley YM, Marshall DJ (2008) Offspring size plasticity in response to intraspecific competition: an adaptive maternal effect across life-history stages. Am Nat 171: 225–237. 18197775 10.1086/524952

[11] Lampert W (1994) Phenotypic plasticity of the filter screens in Daphnia: Adaptation to low-food environment. Limnol Oceanogr 39(5): 997–1006.

[12] Kooijman SALM (2010) Dynamic energy budget theory for metabolic organization. Cambridge University Press: 514p.

[13] Hazlerigg CRE, Lorenzen K, Thorbek P, Wheeler JR, Tyler CR (2012) Density-dependent processes in the life history of fishes: Evidence from laboratory populations of zebrafish *Danio rerio* . PLoS ONE 7(5): e37550. 22655056 10.1371/journal.pone.0037550 PMC3360039

[14] Preuss TG, Hammers-Wirtz M, Hommen U, Rubach MN, Ratte HT (2009) Development and validation of an individual based *Daphnia magna* population model: The influence of crowding on population dynamics. Ecol Model 220: 310–329.

[15] Brand GW (1985) Effect of crowding on larval viability in *Tisbe holothuriae* (Copepoda: Harpacticoida). Mar Biol 88: 67–72.

[16] Post JR, Johannes MRS, Mcqueen DJ (1997) Evidence of density dependent cohort splitting in age-0 yellow perch, (*Perca flavescens*): potential behavioural mechanisms and population-level consequences. Can J Fish Aquat Sci 54: 867–875.

[17] Seitz A (1984) Are there allelopathic interactions in zooplankton? Laboratory experiments with Daphnia. Oecologia 62: 94–96. 28310745 10.1007/BF00377380

[18] Goser B, Ratte HT (1994) Experimental evidence of negative interference in *Daphnia magna* . Oecologia 98: 354–361. 28313912 10.1007/BF00324224

[19] Kirk KL (1998) Enrichment can stabilize population dynamics: autotoxins and density dependence. Ecology 79: 2456–2462.

[20] Moe JS, Kristoffersen AB, Smith RH, Stenseth NC (2005) From patterns to processes and back: analyzing density-dependent responses to an abiotic stressor by statistical and mechanistic modeling. Proc Roy Soc Lond B 272: 2133–2142. 10.1098/rspb.2005.3184 PMC1559955 16191626

[21] Semlitsch RD, Bridges CM, Welch AM (2000) Genetic variation and a fitness tradeoff in the tolerance of gray treefrog (Hyla versicolor) tadpoles to the insecticide carbaryl. Oecologia 125: 179–185. 24595829 10.1007/s004420000443

[22] Lampert W (2006) Daphnia: model herbivore, predator and prey. Polish J Ecol 54: 607–620.

[23] Dodson SI, Hanazato T (1995) Commentary on effects of anthropogenic and natural organic-chemicals on development, swimming behavior, and reproduction of Daphnia, a key member of aquatic ecosystems. Environ Health Perspect 103: 7–11. 10.1289/ehp.95103s47 PMC1519265 7556027

[24] McCauley E, Murdoch WW, Nisbet RM, Gurney WSC (1990) The physiological ecology of Daphnia: Development of a model of growth and reproduction. Ecology 71(2): 703–715.

[25] Wogram J, Liess M (2001) Rank ordering of macroinvertebrate species sensitivity to toxic compounds by comparison with that of Daphnia magna. Bull. Environ. Contam Toxicol 67(3): 360–367. 10.1007/s001280133 11479665

[26] Guisande C, Gliwicz ZM (1992) Egg size and clutch size in two Daphnia species grown at different food levels. J Plankton Res 14 (7): 997–1007.

[27] Plath K (1998) Adaptive feeding behavior of *Daphnia magna* in response to short-term starvation. Limnol Oceanogr 43(4): 593–599.

[28] Pop M (1991) Mechanisms of the filtering area adaptation in *Daphnia* . Hydrobiologia 225: 169–176.

[29] Burns CW (1995) Effects of crowding and different food levels on growth and reproductive investment of Daphnia. Oecologia 101(2): 234–244. 28306796 10.1007/BF00317289

[30] Burns CW (2000) Crowding-induced changes in growth, reproduction and morphology of Daphnia. Freshwater Biol 43: 19–29.

[31] Goser B (1997) Dichteabhängige Änderungen der Entwicklung und Reproduktion bei Cladoceren. Shaker, Aachen: 210p.

[32] Cleuvers M, Goser B, Ratte HT (1997) Life-history shift by intraspecific interaction in *Daphnia magna*: change in reproduction from quantity to quality. Oecologia 110: 337–345. 28307222 10.1007/s004420050167

[33] Sousa T, Domingos T, Kooijman SALM (2008) From empirical patterns to theory: a formal metabolic theory of life. Philos T Roy Soc 363: 2453–2464. 10.1098/rstb.2007.2230 PMC2606805 18331988

[34] Kooijman SALM, Sousa T, Pecquerie L, Van der Meer J, Jager T (2008) From food-dependent statistics to metabolic parameters, a practical guide to the use of dynamic energy budget theory. Biol Rev 83: 533–552. 19016672 10.1111/j.1469-185X.2008.00053.x

[35] Martin BT, Jager T, Preuss TG, Nisbet R, Grimm V (2013) Predicting population dynamics from the properties of individuals: a cross-level test of Dynamic Energy Budget theory. Am Nat 181: 506–519. 23535615 10.1086/669904

[36] Gergs A, Jager T (2014). Body size mediated starvation resistance in an insect predator. J Anim Ecol DOI: –10.1111/1365–2656.12195 10.1111/1365-2656.12195 24417336

[37] Jager T (2012) Making sense of chemical stress application of dynamic energy budget theory in ecotoxicology and stress ecology. E-book available: http://www.debtox.info/book.php: Version 1.0, 14 June 2013.

[38] van der Meer J (2006) An introduction to Dynamic Energy Budget (DEB) models with special emphasis on parameter estimation. J Sea Res 56: 85–102.

[39] Nisbet RM, Muller EB, Lika K, Kooijman SALM (2000) From molecules to ecosystems through dynamic energy budget models. J Anim Ecol 69: 913–926.

[40] Álvarez OA, Jager T, Marco Redondo E, Kammenga JE (2006) Physiological modes of action of toxic chemicals in the nematode *Acrobeloides nanus* . Environ Toxicol Chem 25: 3230–3237. 17220093 10.1897/06-097r.1

[41] Gophen M, Geller W (1984) Filter mesh size and food particle uptake by Daphnia. Oecologia 64: 408–412. 28311458 10.1007/BF00379140

[42] Jager T, Selck H (2011) Interpreting toxicity data in a DEB framework: A case study for nonylphenol in the marine polychaete *Capitella teleta* . J Sea Res 66: 456–462.

[43] Jager T, Barsi A, Hamda NT, Matrin BT, Zimmer E, et al (2013) Dynamic energy budgets in population ecotoxicology: Applications and outlook. Ecol. Model. DOI: 10.1016/j.ecolmodel.2013.06.024

[44] Nelder JA, Mead R (1965) A simplex-method for function minimization. Comp J 7: 308–313.

[45] Jager T, Albert C, Preuss TG, Ashauer R (2011) General unified threshold model of survival – a toxicokinetic-toxicodynamic framework for ecotoxicology. Environ Sci Technol 45: 2529–2540. 21366215 10.1021/es103092a

[46] Nash JE, Sutcliffe JV (1970) River flow forecasting through conceptual models: Part 1. A discussion of principles. J Hydrol 10(3): 282–290.

[47] Siehoff S, Hammers-Wirtz M, Strauss T, Ratte HT (2009) Periphyton as alternative food source for the filter-feeding cladoceran *Daphnia magna* . Freshwater Biol 54: 15–23.

[48] Sokull-Kluettgen B (1998) Die kombinierte Wirkung von Nahrungsangebot und 3,4-Dichloranilin auf die Lebensdaten von zwei nahverwandten Cladocerenarten, Daphnia magna und Ceriodaphnia quadrangula. Shaker, Aachen: 110p.

[49] Bohrer RN, Lampert W (1988) Simultaneous measurement of the effect of food concentration on assimilation and respiration in Daphnia magna Straus. Funct Ecol 2: 463–471.

[50] Hammers-Wirtz M, Ratte HT (2003) Erstellung eines Gutachtens zur Entwicklung eines Verfahrensvorschlages für einen Daphnia Multi Generation Test. Federal Environmental Agency Germany, FKZ 360 03 024.

[51] Elendt BP, Bias WR (1990) Trace nutrient deficiency in *Daphnia magna* cultured in standard medium for toxicity testing – effects of the optimization of culture conditions on life-history parameters of *Daphnia magna* . Water Res 24: 1157–1167.

[52] McMahon JW, Rigler FH (1963) Mechanisms regulating feeding rate of *Daphnia magna* Straus. Can J Zoolog 41(2): 321–327.

[53] Rigler FH (1961) The relation between concentration of food and feeding rate of *Daphnia magna* Straus. Can J Zoolog 39: 857–868.

[54] Repka S, Veen A, Vijverberg J (1999) Morphological adaptations in filtering screens of *Daphnia galeata* to food quantity and food quality. J Plankton Res 21(5): 971–989.

[55] Lampert W, Brendelberger H (1996) Strategies of phenotypic low-food adaptation in Daphnia: Filter screens, mesh sizes, and appendage beat rates. Limnol Oceanogr 41(2): 216–223.

[56] Bradley MC, Perrin N, Calow P (1991) Energy allocation in the cladoceran *Daphnia magna* Strauss, under starvation and refeeding. Oecologia 86: 414–418. 28312930 10.1007/BF00317610

[57] Hayward RS, Gallup DN (1976) Feeding, filtering and assimilation in *Daphnia schoedleri* Sars as affected by environmental conditions. Arch Hydrobiol 77: 139–163.

[58] Helgen JC (1987) Feeding rate inhibition in crowded *Daphnia pulex* . Hydrobiologia 154: 113–119.

[59] Matveev V (1993) An investigation of allelopathic effects of daphnia. Freshwater Biol 29 (1): 99–105.

[60] Sousa T, Domingos T, Poggiale JC, Kooijman SALM (2012) Dynamic energy budget theory restores coherence in biology Philos T Roy Soc. 365: 3413–3428. 10.1098/rstb.2010.0166 PMC2981977 20921042

[61] Brooks JL, Dodson SI (1965) Predation, body size, and the composition of the plankton. Science 150: 28–35. 17829740 10.1126/science.150.3692.28

[62] Threlkeld ST (1976) Starvation and the size structure of zooplankton communities. Freshwater Biol 6: 489–496.

[63] Renault D, Hance T, Vannier G, Vernon P (2003) Is body size an influential parameter in determining the duration of survival at low temperatures in *Alphitobius diaperinus* Panzer (Coleoptera: Tenebrionidae)? J Zool 259: 381–388.

[64] Lehmann T, Dalton R, Kim EH, Dahl E, Diabate A, et al (2006) Genetic contribution to variation in larval development time, adult size, and longevity of starved adults of *Anopheles gambiae* . Infect Genet Evol 6: 410–416. 16524787 10.1016/j.meegid.2006.01.007

[65] Kearney M, Simpson SJ, Raubenheimer D, Helmuth B (2010) Modelling the ecological niche from functional traits. Phil Trans R Soc B 365: 3469–3483. 20921046 10.1098/rstb.2010.0034 PMC2981966

[66] Urabe J, Sterner RW (2001) Contrasting effects of different types of resource depletion on life-history traits in Daphnia. Funct Ecol 15: 165–174.

[67] Nisbet RM, McCauley E, Johnson LR (2010) Dynamic energy budget theory and population ecology: lessons from Daphnia. Philos T Roy Soc B 365: 3541–3552. 10.1098/rstb.2010.0167 PMC2981978 20921052

[68] Stearns SC (1976) Life-History Tactics: A Review of the Ideas. Q Rev Biol 51: 3–47. 778893 10.1086/409052

[69] Boggs CL (1992) Resource allocation: exploring connections between foraging and life history. Funct Ecol 6: 508–518.

[70] McCauley E, Nelson WA, Nisbet RM (2008) Small-amplitude cycles emerge from stage-structured interactions in Daphnia-algal systems. Nature 455(7217): 1240–1243. 18972019 10.1038/nature07220

[71] Sibly RM (1999) Efficient experimental designs for studying stress and population density in animal populations. Ecol Appl 9(2): 496–503.

[72] Goulden CE, Hornig LL (1980) Population oscillations and energy reserves in planktonic cladocera and their consequences to competition. Proc Natl Acad Sci USA 77: 1716–1720. 16592788 10.1073/pnas.77.3.1716 PMC348568

[73] Boersma M (1997) Offspring size and parental fitness in Daphnia magna. Evolutionary Ecology 11: 439–450.

[74] Hobæk A, Larsson P (1990) Sex determination in Daphnia magna. Ecology 71 (6): 2255–2268.

[75] Kleiven OT, Larsson P, Hobæk A (1992) Sexual reproduction in Daphnia magna requires three stimuli. Oikos 65: 197–206.

[76] Chamberlin TC (1931) The method of multiple working hypotheses. J Geol 39(2): 155–165.

[77] Burnham KP, Anderson DR (2004) Multimodel inference – understanding AIC and BIC in model selection. Sociological Methods and Research 33(2): 261–304.

[78] Forster MR (2000) Key concepts in model selection: Performance and generalizability. J Math Psychol 44: 205–231. 10733865 10.1006/jmps.1999.1284

[79] Browne MW (2000) Cross-validation methods. J Math Psychol 44: 108–132. 10733860 10.1006/jmps.1999.1279

[80] Begon M, Townsend CR, Harper JL (2005) Ecology: from individuals to ecosystems. Wiley-Blackwell, New Jersey: 752p.

[81] Ricklefs RE, Miller G (1999) Ecology. W.H. Freeman, New York: 822p.

[82] Bassar RD, Lopez-Sepulcre A, Reznick DN, Travis J (2013) Experimental evidence for density-dependent regulation and selection on Trinidadian guppy life histories. Am Nat 181(1): 25–38. 23234843 10.1086/668590

[83] Kendall BE, Briggs CT, Murdoch WW, Turchin P, Ellner SP, et al (1999) Why do populations cycle? A synthesis of statistical and mechanistic modeling approaches. Ecology 80: 1789–1805.

