# Supplementary material for: Double Trouble at High Density: Cross-Level Test of Resource-Related Adaptive Plasticity and Crowding-Related Fitness
Source: PLoS One. 2014 Mar 13;9(3):e91503. doi: 10.1371/journal.pone.0091503 (PMC3953409; doi:10.1371/journal.pone.0091503)
Supplement: Supporting Information S1 — Model description and source code. (PDF) [file pone.0091503.s001.pdf]

Double trouble at high density: cross-level test of resource-related adaptive plasticity and crowding-related fitness

André Gergs, Thomas G. Preuss and Annemette Palmqvist

Supporting information S1

Model description

The model description follows the ODD (Overview, Design concepts, Details) protocol for describing individual-based models [1, 2].

1. Purpose

The purpose of the model is the analysis and prediction of the population dynamics of *Daphnia magna,* with special emphasis on competition for food and stress that might occur at high population densities independent of food availability, a phenomenon that is referred to as crowding.

2. Entities, state variables and scales

The model is organised on three hierarchical levels that are represented by three corresponding entities or rather objects in the model, i.e. ecosystem, population and individual. The highest level is the ecosystem, which includes environmental properties such as ecosystem volume and food dynamics, i.e. the addition of food and the actual food concentration. In its current state, the model is restricted to an ambient temperature of 20°C, a 16:8h (light:dark) photoperiod and environments that scale up to a few litres. The only state variables of the ecosystem are therefore the volume and the total organic carbon (C) available as a food source for daphnids [mgC L^{-1}]. The food density is subject to change due to the addition of food on fixed dates and its depletion as a result of daphnid feeding activity. The second entity, the population, has a book-keeping function within the model as it monitors the abundances. It is represented by two state variables: the population density [# L^{-1}] and the population feeding rate [mgC h^{-1}], which is the summed feeding rate of all daphnid individuals. The population hosts a number of *D. magna* individuals that are modelled by their life cycles. Changes in population density are determined by individual birth and death events. Each

daphnid individual is represented by formulations of the dynamic energy budget (DEB) theory [3, 4, 5] to describe its acquisition and energy use. The current model is based on the standard scaled DEB model as published by Kooijman et al. [5], where reserves and maturity are scaled with maximum assimilation rate per unit surface area. This removes the dimensions of energy or mass from the system. *Daphnia* individuals are characterised by five primary state variables: (1) structural length L [mm] relates to physical length and determines actual feeding rates and costs for maintaining body functions; (2) the amount of scaled reserve U_E [mm^2 d] serves as an energy storage and provides a link between food assimilation and allocation of energy within the organism; (3) the scaled maturity U_H [mm^2 d] regulates individual development and stage transitions are initiated by fixed maturity levels: $U_H = U_H^b$ for birth and $U_H = U_H^p$ at puberty; (4) the scaled reproduction buffer U_R [mm^2 d] represents the energy that is available for reproduction. During reproduction events, the scaled reproduction buffer is converted into eggs. (5) The vital state of the individual is based on a probability for survival S [-] and determines whether an individual is alive or dead and whether the individual should be included in the population count or removed from the population object. In addition to primary state variables, a number of intermediate outputs or states are necessary within each individual for updating primary states. These intermediate outputs are described in the submodel section below. An overview of state variables and outputs is provided in Table S1. Description of parameters is given in the main text.

3. Process overview and scheduling

The model proceeds in discrete time steps: *Daphnia* state variables and intermediate outputs are updated and growth and reproduction are calculated on a daily basis, whereas feeding and assimilation are calculated in hourly steps. Each hour, feeding rates of individual daphnids are summed, to derive a population feeding rate. If the (potential) population feeding rate exceeds the amount of food available in the environment, the time interval is reduced and population feeding rates are calculated in iterations of an hour, e.g. for an iteration number of four, the population feeding rate will be calculated in steps of quarter hours. At each time step, the environmental food concentration is reduced by the population feeding rate.

Within a single day, model processes are executed in the following order: food is added to the ecosystem in accordance with experimental scenarios; food density in the environment is calculated per hour based on the experimental food scenario and *Daphnia* feeding rates in the preceding time step. Thereafter, feeding is calculated for individual *Daphnia* and hourly feeding rates are summed,

to obtain the daily food intake. At the same time, assimilation, i.e. the uptake of mass or energy into the body, is calculated as a function of the food availability and assimilates are converted into the reserve. One fraction of the reserve κ is used for somatic maintenance and growth, whereas 1- κ contributes to maturity maintenance and maturation or, after puberty, to the reproduction buffer. In mature daphnids, the accumulated reproduction buffer is converted into embryos and embryonic growth as well as embryonic maturation modelled thereafter. On reaching scaled maturity at birth, U_H^b embryos are released into the environment and a new brood is created. Each day, individual daphnids have a probability of dying, which is proportional to damage that is either caused by ageing or starvation. At the end of each simulated day, dead individuals are removed and newborns added to the population count.

4. Design concepts

Emergence. Individual life history traits as well as population dynamics and population structure emerge from metabolic organisation and intraspecific competition of daphnid individuals for food.

Adaptation. *Daphnia* individuals adapt life history processes to conditions of low food availability by increasing filtration rate, increasing assimilation efficiency and changing the fraction of reserve that is allocated to soma κ. If, during starvation, the scaled reserve density, *e*, drops below a non-growth boundary, reserves are mobilised to pay somatic maintenance costs only; at the same time, growth as well as the allocation of reserves to the maturation or reproduction buffer cease. Details on adaptation and crowding stress submodels are given in the main text.

Sensing. Individual daphnids sense the population density and the environmental food density.

Interactions. Indirect interactions via competition for food and space are included in the model, whereas direct interactions are not considered.

Stochasticity. There are three sources of stochasticity included in the model. First, surface-area-specific filtration rate is multiplied by a random number drawn from a normal distribution (with a mean of one and standard deviation of 0.231, see [6]) and this is assigned to individual daphnids at birth. Second, mortality is assumed to be a probabilistic process. For each individual, a survival probability is calculated, based on modules for ageing and starvation. An individual dies if the survival probability exceeds a random number for survival (drawn from a uniform distribution between 0 and 1), which is assigned to each daphnid at birth. The third source of stochasticity is related to the composition of the start population (see initialisation section).

Observation. During the time-course of simulations, the total number of daphnids and the abundance within three size classes (small, medium, large) are observed. In the original experiments, size classes were obtained by sieving daphnids through mesh filters of various sizes. Preuss et al. [6] calculated the range of body length per size class based on the mesh diameter, multiplied by a length-to-width factor for daphnids of 1.6, assuming that daphnids passed through the mesh with their smallest dimensions. However, Martin et al. [7] found that a conversion factor of 1.6 overestimates actual body sizes. We therefore empirically determined size classes by sieving daphnids through the meshes used in the original study and subsequently measured daphnid body length excluding the spine, for the medium and large size classes (n = 100 each). We use the lower 95% confidence interval of measurements determined for medium and large size classes as lower class boundaries respectively, corresponding to size classes of: <1.25 mm (small), ≥1.25 – <2.1 mm (medium) and ≥2.1 mm (large). These values are close to the size classes used by Martin et al. [7]. To compare the composition of simulated populations and experimental results, we relate the actual body length measure L_w to structural length by using a shape correction coefficient δ_M which translates physical length to volumetric length:

$$L_W = \frac{L}{\delta_M}$$

For Monte-Carlo simulations, the mean and 95% confidence interval are calculated for total abundance and abundance within each size class based on values recorded each day for each simulation run.

5. Initialisation

Initialisation of model simulations is based on the experimental conditions they are supposed to represent, i.e. the volume of the environment (test vessel) as well as the abundance and the composition of the population at the start of the experiment. The simulations start with five neonate daphnids, less than one day old, and in addition, three adult daphnids, between two and three weeks old. To mimic these initial conditions, we simulate growth, maturation and reproduction as well as survival, until each daphnid reaches its respective age, starting with a body length at birth of 0.9 mm. For adults, a random age at simulation starts between 14 and 21 and is drawn from a uniform distribution. To represent food availability under culture conditions, we used a fixed scaled functional response of f = 0.7 (see submodel section), which fits growth measured under culture conditions well (unpublished result). Analogous to the experiments, adult daphnids are assumed to

be egg-carrying, one with new egg (embryo maturity level U_H = 0), and two adults with further-developed eggs (embryo maturity level randomly drawn from a uniform distribution between 0 and 0.005).

6. Input data

The amount of food added to the system is included as input data to the model, representing the major external driving process in the laboratory setting. In accordance with the experimental scenario, food is added to the modelled environment daily on weekdays, with the amount tripled on fridays and no food is added during weekends. We used the two different scenarios of low food (0.5 mg total organic carbon (C) per day and population) and high food (1.3 mg C per day and population).

7. Submodels

Feeding and assimilation. The food ingestion rate is assumed to follow a functional response type II [8, 9]:

$$J_X = J_{Xm} \frac{X}{X_K + X}$$

Where J_{Xm} is the maximum ingestion rate for a filtration rate and is determined by the incipient limiting level [10]:

$$J_{Xm} = ill \, \{F_X\} \, L^2$$

Change in environmental food density X is due to the summed food ingestion of all daphnid individuals i:

$$X(t) = X(t-1) - \sum_i J_{X_i}$$

Ingested food is assimilated with a certain efficiency p_x. The fraction of food that is not assimilated by the daphnid is excreted from the gut. In the model, we do not follow possible recycling of faeces and assume that these are immediately removed from the system. The assimilation rate is determined by the ingestion rate and the assimilation efficiency:

$$p_A = p_X \, J_X$$

Although DEB theory makes the assumption that food is assimilated at a constant efficiency, there is some evidence that daphnids strive to increase assimilation efficiency at low food concentrations [11], probably by increasing gut residence times. In accordance with Rinke and Vijverberg [12], we assume the assimilation efficiency to be dependent on external food concentration:

$$p_X = p_{X_{max}} - \frac{\left(p_{X_{max}} - p_{X_{min}}\right) X}{X_k + x}$$

The scaled functional response then becomes a function of the summed realised assimilation per day and the maximum daily assimilation rate:

$$f = \frac{\sum_t p_{A_t}}{\{p_{Am}\} L^2}$$

Reserve dynamic. The scaled amount of reserve U_E in an individual is determined by the difference in scaled assimilation $f\,L^2$ and the mobilisation flux *Sc*, with the onset of feeding activity after birth ($U_H \geq U_H^b$):

$$\frac{dU_E}{dt} = \begin{cases} -S_c & if\ U_H < U_H^b \\ f L^2 - S_c & if\ U_H \geq U_H^b \end{cases}$$

Where under growth conditions ($e > l$, with $l = \frac{L\ g k_M}{v\ e}$), the mobilisation flux S_c is:

$$Sc = L^2 \frac{g\ e}{g + e}\left(1 + \frac{k_M L}{v}\right)$$

Whereby the scaled reserve density, *e*, is given by:

$$e = v \frac{U_E}{L^3}$$

At low environmental food density, the reserve drops below a non-growth boundary ($e \leq l$). Thereafter, we calculate S_c under the assumption that daphnids do not shrink during starvation and that the reserve is used to pay somatic maintenance costs only:

$$Sc = \frac{\kappa\ k_M g L^3}{v}$$

Growth. Growth is considered to be the net result of mobilised reserve that is allocated to soma κ (included in the compound parameter $g = \frac{[E_G]}{\kappa[E_m]}$ with $[E_m] = \frac{\{p_{Am}\}}{v}$), minus the costs for somatic maintenance, thus, the change in structural length is given by:

$$\frac{dL}{dt} = \frac{1}{3L^2}\left(\frac{v}{g}S_c - k_M L^3\right)$$

If food is scarce and the amount of reserve does not allow the animal to grow ($e \leq l$), then $\frac{dL}{dt} = 0$. The animal dry weight includes structure and the mass of reserve and is calculated as a function of the cubed structural length:

$$W_d = d_{Vd}L^3(1 + \omega_d e)$$

Maturation and Reproduction. A fraction of the mobilised reserves (1- κ) is allocated to maturation or reproduction. The change in scaled maturity is given by:

$$\frac{dU_H}{dt} = \begin{cases} (1-\kappa)S_c - k_J U_H & if\ U_H < U_H^p \\ 0 & if\ U_H \geq U_H^p \end{cases}$$

Once puberty is reached ($U_H \geq U_H^p$), the maturity level is fixed and mobilised reserves (1- κ) are allocated to the reproduction buffer:

$$\frac{dU_R}{dt} = \begin{cases} 0 & if\ U_H < U_H^p \\ (1-\kappa)S_c - k_J U_H^p & if\ U_H \geq U_H^p \end{cases}$$

At certain time steps, the reproduction buffer is converted into a number of embryos *R* with a fixed reproduction efficiency k_r. The ratio of the energy that can efficiently be used for producing embryos and the cost of a single embryo U_E^0 determines the clutch size:

$$R = \frac{k_r U_R}{U_E^0}$$

As only whole embryos can be produced, the clutch size is rounded to the lower integer and the remaining energy is kept in the reproduction buffer for the next reproductive event. The DEB theory assumes that initial embryonic reserves are such that the embryo will hatch at the same reserve density as the mother experienced at embryo production. One way to determine the initial reserve would be to test different embryonic reserve levels and to simulate embryo development until the respective maternal reserve density is observed. As this method is computationally expensive, we

use an approximation and model embryonic growth ($L(0) = 0.00001$) and maturation ($U_H(0) = 0$) by assuming a fixed scaled reserve density, *e* (set to the maternal *e* at embryo formation). When embryos surpass scaled maturity at birth ($U_H > U_H^b$), they are released by the mother and a new clutch is formed. At birth, the embryonic reserve level is re-calculated by $U_E = \frac{eL^3}{v}$. Consequently, the reserve level does not change during embryonic development and U_E^0 is only used to determine the clutch size. More effective procedures for the calculation of $U_E(0)$ are given by Kooijman [13].

Survival. Two causes of death are considered in the model: ageing and starvation. The basic idea behind the effect of ageing on survival is that damage-inducing compounds are accumulated at a rate proportional to the rate of energy mobilisation and each of the damage-inducing compounds is copied also at a rate proportional to energy mobilization [4]. The process of damage production can be summarised as ageing acceleration *q*:

$$\frac{dq}{dt} = \left(q \frac{L^3}{\left(\frac{v}{gk_M}\right)^3} s_G + h_a \right) e \left(\frac{v}{L} - \frac{3dL}{L\,dt}\right) - \frac{3dL}{L\,dt} q$$

The resulting hazard rate due to ageing is:

$$\frac{dh}{dt} = q - \frac{3dL}{L\,dt} h$$

The above formulation for ageing assumes that daphnids live longer at low food levels compared to higher ones. However, at some point the food level does not allow the full coverage of maintenance costs and starvation occurs. We assume that at low reserve density, additional damage is caused by some type of physiological disturbance. Modified from Jager et al. [14], this scaled damage (D^*) is assumed to accumulate proportional to one minus the scaled reserve density, and is repaired in proportion to the actual damage level with a fixed rate:

$$\frac{dD^*(t)}{\mathrm{d}t} = k_d(\,(1 - e) - D^*(t))$$

To link scaled damage to the survival probability, we use the concept of individual tolerance, a special case of the general unified threshold model of survival [14]. In individual tolerance models, the threshold for survival follows a frequency distribution within a population and death is

instantaneous for an organism when damage exceeds the individual survival threshold. In accordance with Nyman et al. [15], the threshold is drawn from a log-logistic cumulative distribution function:

$$F_s(t) = \frac{1}{1 + \left(\frac{\max\limits_{0<\tau<t} D^*(\tau)}{\alpha}\right)^{-\beta}}$$

For a discussion of the starvation model see [16].

Survival probability at time *t* is calculated based on the individual survival threshold for starvation and the hazard rate for ageing at time t:

$$S(t) = \big(1 - F_s(t)\big)\ \exp\,(-h(t))$$

A *Daphnia* individual is considered as being dead when the survival probability is below a random number for survival that is drawn from a uniform distribution between 0 and 1 and assigned to the individual at birth.

8. Implementation

The model is implemented in Delphi Xe2 (Embarcadero Technologies San Francisco, USA, 2011) and is based on discretised forms of the differential equation submodels as described above.

References

1. Grimm V, Berger U, Bastiansen F, Eliassen S, Ginot V et al. (2006) A standard protocol for describing individual-based and agent-based models. Ecol Model 198: 115-126.

2. Grimm V, Berger U, DeAngelis DL, Polhill, G, Giske J et al. (2010) The ODD protocol: a review and first update. Ecol Model 221: 2760-2768.

3. Kooijman SALM (2001) Quantitative aspects of metabolic organization: a discussion of concepts. Philos T Ro Soc 356: 331–349.

4. Kooijman SALM (2010) Dynamic energy budget theory for metabolic organization. Cambridge University Press.

5. Kooijman SALM, Sousa T, Pecquerie L, Van der Meer J, Jager T (2008) From food-dependent statistics to metabolic parameters, a practical guide to the use of dynamic energy budget theory. Biol Rev 83: 533-552.

6. Preuss TG, Hammers-Wirtz M, Hommen U, Rubach MN, Ratte HT (2009) Development and validation of an individual based Daphnia magna population model: The influence of crowding on population dynamics. Ecol Model 220: 310-329.

7. Martin BT, Jager T, Preuss TG, Nisbet R, Grimm V (2013) Predicting population dynamics from the properties of individuals: a cross-level test of Dynamic Energy Budget theory. Am Nat 181: 506-519.

8. Holling CS (1959a) The components of predation revealed by a study of small-mammal predation of the European pine sawfly. Can Entomol 91: 293-320.

9. Holling CS (1959b) Some characteristics of simple types of predation and parasitism. Can Entomol 91: 385-398.

10. McMahon JW, Rigler FH (1963) Mechanisms regulating feeding rate of Daphnia magna Straus. Can J Zool 41(2): 321-327.

11. Urabe J, Watanabe Y (1990) Influence of food density on respiration rate of two crustacean plankters. Daphnia galeata and Bosmina longirostris. Oecologia 82: 362-368.

12. Rinke K, Vijverberg J (2005) A model approach to evaluate the effect of temperature and food concentration on individual life-history and population dynamics of Daphnia. Ecol Model 186(3): 326–344.

13. Kooijman SALM (2009) What the egg can tell about its hen: embryonic development on the basis of dynamic energy budgets. J Math Biol 58(3): 377-394.

14. Jager T, Albert C, Preuss TG, Ashauer R (2011) General unified threshold model of survival - a toxicokinetic-toxicodynamic framework for ecotoxicology. Environ Sci Technol 45: 2529-2540.

15. Nyman AM, Schirmer K, Ashauer R (2012). Toxicokinetic-toxicodynamic modelling of survival of Gammarus pulex in multiple pulse exposures to propiconazole: model assumptions, calibration data requirements and predictive power. Ecotoxicology 21: 1828-1840.

16. Gergs A, Jager T (2014). Body size mediated starvation resistance in an insect predator. J Anim Ecol DOI: 10.1111/1365-2656.12195.

Table S1: Variables and intermediate outputs of the model.

Symbol	Description	Unit
D^*	Scaled damage	[-]
d_P	Population density	[# mL^{-1}]
e	Scaled reserve density	[-]
$[E_m]$	Maximum reserve density	[mg mm^{-3}]
F_s	Individual tolerance threshold	[-]
f	Scaled functional response	[-]
g	Energy investment ratio	[-]
h	Hazard rate	[d^{-1}]
J_X	Ingestion rate	[mg h^{-1}]
J_{Xm}	Maximum ingestion rate	[mg h^{-1}]
L	Volumetric structural length	[mm]
L_w	Actual body length	[mm]
p_A	Surface-area-specific	[mg mm^{-2} h^{-1}]
P_X	Assimilation efficiency	[-]
R	Reproductive rate	[# d^{-1}]
S	Survival probability	[-]
s_d	Stress function for crowding	[-]
S_C	Scaled catabolic flux	[mm^2]
U_E	Scaled reserve level	[mm^2 d]
U_H	Scaled maturity level	[mm^2 d]
U_R	Scaled reproduction buffer	[mm^2 d]
q	Ageing acceleration	[d^{-2}]
W_d	Dry weight of the organism	[mg]
X	Food density in the environment	[mg mL^{-1}]

Source code

In the following section the source code of the model, implemented in Delphi XE2, is given. The full implementation including an executable file can be obtained from the authors on request. Please also note, that the executable file does not require the installation of any software, and can be used independent of the Delphi programming environment.

```
{:::::::::::::::::::::::::::::::::::::::::::::::::::::::::::::::::::::::::::::::::::::::::::::::::::::::::::::::::::::::::::::::::::::::::::::
:::::::::::::::::::::::::::::::::::::::::::::::::::::::::::::::::::::::::::::::::::::::::::::::::::::::::::::::::::::::::::::::::::::::::::::::
      In this unit settings on the user interface are handled and simulations started
:::::::::::::::::::::::::::::::::::::::::::::::::::::::::::::::::::::::::::::::::::::::::::::::::::::::::::::::::::::::::::::::::::::::::::::::
::::::::::::::::::::::::::::::::::::::::::::::::::::::::::::::::::::::::::::::::::::::::::::::::::::::::::::::::::::::::::::::::::::::::::::::}
unit Start;

interface

uses
  Windows, Messages, SysUtils, Variants, Classes, Graphics, Controls, Forms, Dialogs, Menus, ComCtrls, ExtCtrls,
  Ecosystem, Environment, Population, Individual, Output,LoadData;

//---declarations of TForm--------------------------------------------------------------------
type
  TForm_Start = class(TForm)
    MainMenu1:                          TMainMenu;
    Start1:                             TMenuItem;
    SingleSimulation1:                  TMenuItem;
    MonteCarloSimulation1:              TMenuItem;
    Load1:                              TMenuItem;
    Save1:                              TMenuItem;
    TB_Setting:                         TTabControl;
    Populationdata1:                    TMenuItem;
    Populationresults1:                 TMenuItem;

    procedure TB_SettingChange          (Sender: TObject);
    procedure open1Click                (Sender: TObject);
    procedure close1Click               (Sender: TObject);
    procedure SingleSimulation1Click    (Sender: TObject);
    procedure MonteCarloSimulation1Click (Sender: TObject);
    procedure hide1Click                (Sender: TObject);
    procedure show1Click                (Sender: TObject);
    procedure Populationdata1Click      (Sender: TObject);
    procedure Populationresults1Click   (Sender: TObject);
  end;

var
  Form_Start: TForm_Start;

implementation
{$R *.dfm}
{xxxxxxxxxxxxxxxxxxxxxxxxxxxxxxxxxxxxxxxxxxxxxxxxxxxxxxxxxxxxxxxxxxxxxxxxxxxxxxxxxxxx
               Main menu procedures: Simulation
xxxxxxxxxxxxxxxxxxxxxxxxxxxxxxxxxxxxxxxxxxxxxxxxxxxxxxxxxxxxxxxxxxxxxxxxxxxxxxxxxxxxxx}
// if Monte-Carlo simulation is selected:
procedure TForm_Start.MonteCarloSimulation1Click(Sender: TObject);
begin
    Ecosystem.Form_Ecosystem.RG_Simtype.ItemIndex:=1;
    Ecosystem.Form_Ecosystem.readsettings;
    Ecosystem.Form_Ecosystem.runregularsimulation;
end;
```

```
//if single simulation run is selected:
procedure TForm_Start.SingleSimulation1Click(Sender: TObject);
begin
    Ecosystem.Form_Ecosystem.RG_Simtype.ItemIndex:=0;
    Ecosystem.Form_Ecosystem.readsettings;
    Ecosystem.Form_Ecosystem.runregularsimulation;
end;

{xxxxxxxxxxxxxxxxxxxxxxxxxxxxxxxxxxxxxxxxxxxxxxxxxxxxxxxxxxxxxxxxxxxxxxxxxxxxxxxxxxx
              Main menu procedures: Load
xxxxxxxxxxxxxxxxxxxxxxxxxxxxxxxxxxxxxxxxxxxxxxxxxxxxxxxxxxxxxxxxxxxxxxxxxxxxxxxxxxxx}
procedure TForm_Start.Populationdata1Click(Sender: TObject);
begin
 Form_Load.loadpopdata;
 Form_Load.showloaddata;
end;

procedure TForm_Start.Populationresults1Click(Sender: TObject);
begin
  Output.Form_Output.savepopresult;
end;

{xxxxxxxxxxxxxxxxxxxxxxxxxxxxxxxxxxxxxxxxxxxxxxxxxxxxxxxxxxxxxxxxxxxxxxxxxxxxxxxxxxx
              Main menue procedures: Output
xxxxxxxxxxxxxxxxxxxxxxxxxxxxxxxxxxxxxxxxxxxxxxxxxxxxxxxxxxxxxxxxxxxxxxxxxxxxxxxxxxxx}
procedure TForm_Start.show1Click(Sender: TObject);
begin
  Output.Form_Output.Show;
end;

procedure TForm_Start.hide1Click(Sender: TObject);
begin
   Output.Form_Output.hide;
end;

{xxxxxxxxxxxxxxxxxxxxxxxxxxxxxxxxxxxxxxxxxxxxxxxxxxxxxxxxxxxxxxxxxxxxxxxxxxxxxxxxxxx
              Click procedures for setting tabs
xxxxxxxxxxxxxxxxxxxxxxxxxxxxxxxxxxxxxxxxxxxxxxxxxxxxxxxxxxxxxxxxxxxxxxxxxxxxxxxxxxxx}
procedure TForm_Start.open1Click(Sender: TObject);
begin
 TB_Setting.show;
 Form_Ecosystem.Show;
 Form_Environment.hide;
 Form_Population.hide;
 Form_Individual.hide;
 Form_Load.hide;
end;

procedure TForm_Start.close1Click(Sender: TObject);
begin
 TB_Setting.hide;
 Form_Ecosystem.hide;
```

```
 Form_Environment.hide;
 Form_Population.hide;
 Form_Individual.hide;
 Form_Load.hide;
end;

//Shows and hides different forms when selecting tabs:
procedure TForm_Start.TB_SettingChange(Sender: TObject);
begin
 case TB_Setting.TabIndex of
    0:begin Form_Ecosystem.Show;Form_Environment.hide;Form_Population.hide;Form_Individual.hide;      end;
    1:begin Form_Environment.Show; Form_Ecosystem.hide; Form_Population.hide; Form_Individual.hide;   end;
    2:begin Form_Population.Show; Form_Ecosystem.hide; Form_Environment.hide; Form_Individual.hide;   end;
    3:begin Form_Individual.Show; Form_Population.hide; Form_Ecosystem.hide; Form_Environment.hide;   end;
 end;
end;
end.
```

```
{:::::::::::::::::::::::::::::::::::::::::::::::::::::::::::::::::::::::::::::::::::::::::::::::::::::::::::::::::::::::::::::::::::::::::::
::::::::::::::::::::::::::::::::::::::::::::::::::::::::::::::::::::::::::::::::::::::::::::::::::::::::::::::::::::::::::::::::::::::::::::
      This unit handles simulation runs and model output calculations and hosts the population and environment
::::::::::::::::::::::::::::::::::::::::::::::::::::::::::::::::::::::::::::::::::::::::::::::::::::::::::::::::::::::::::::::::::::::::::::
:::::::::::::::::::::::::::::::::::::::::::::::::::::::::::::::::::::::::::::::::::::::::::::::::::::::::::::::::::::::::::::::::::::::::::}
unit Ecosystem;

interface

uses
  Windows, Messages, SysUtils, Variants, Classes, Graphics, Controls, Forms, Dialogs, StdCtrls, ExtCtrls, math,
  Population, Output, Environment, LoadData, LoadEnvData, Toxicant;

//---Declarations for ecosystem settings----------------------------------------
type
  T_eco=record
   //simulation time [d]
   simtime:                              integer;
   //number of simulations
   simnumber:                            integer;
end;

//---Data produced in simulation runs--------------------------------------------
type
  //saves data in an array of array of record per Monte-Carlo Simulation and per day for endpoint calculation in Output unit:
  T_simdata=record
  //Total abundance in size classes/cohorts
  cohort_abundance:                     array of integer;
  //Mean reserve density in size classes/cohorts
  cohort_e:                             array of double;
  //Population feeding rate [mg/(d*pop)]
  popfeedingrate:                       double;
  //food leftover [mgC]
  foodleft:                             double;
end;

//---declarations of TForm-------------------------------------------------------
type
  TForm_Ecosystem = class(TForm)
    RG_Simtype, RG_CI:                               TRadioGroup;
    procedure simulationrun;
    procedure runregularsimulation;
    procedure readsettings;

  public
    eco:                                             T_eco;
    Simdata:                                         array of array of T_simdata;
    pop:                                             T_population;
    env:                                             T_environment;
    endpoint:                                        T_endpoint;
    fooddata:                                        T_fooddata;
    procedure calculatepopendpoints;
```

```
  end;

var
  Form_Ecosystem: TForm_Ecosystem;

implementation
{$R *.dfm}
{xxxxxxxxxxxxxxxxxxxxxxxxxxxxxxxxxxxxxxxxxxxxxxxxxxxxxxxxxxxxxxxxxxxxxxxxxxxxxxxxxx
              Run population simulation
xxxxxxxxxxxxxxxxxxxxxxxxxxxxxxxxxxxxxxxxxxxxxxxxxxxxxxxxxxxxxxxxxxxxxxxxxxxxxxxxxxxxx}
procedure TForm_Ecosystem.simulationrun;
var
m,d:integer;
begin
//run through simulations
  for m := 0 to eco.simnumber - 1 do
    begin
    //creates a population:
     pop:=T_population.create;
     //run through time
        for d := 0 to eco.simtime - 1 do
          begin
           //saves day of simulation in environment:
             env.day:=d;
           //calculates food availability per day by considering leftovers from previous day:
             if d=0 then env.foodavailability[d]:= env.foodtotal[d]
              else env.foodavailability[d]:= env.foodtotal[d]+env.foodleft[d-1];

           //runs through population:
             pop.runthrough (env);
           //computes food leftover on actual day:
             env.foodleft[d]:=env.foodtotal[d]-pop.feedingrate;
             if env.foodleft[d] <0 then   env.foodleft[d]:=0;

           //saves total abundance and size class (cohort) abundance as well as mean reserve density on actual day for actual simulation:
             setlength (simdata[m,d].cohort_abundance, 4);
             setlength (simdata[m,d].cohort_e, 3);
             //total abundance:
             simdata[m,d].cohort_abundance[0]:=pop.poplist.Count;
             //abundance class 1:
             simdata[m,d].cohort_abundance[1]:=pop.sizeclass[0].number;
             //abundance class 2:
             simdata[m,d].cohort_abundance[2]:=pop.sizeclass[1].number;
             //abundance class 3:
             simdata[m,d].cohort_abundance[3]:=pop.sizeclass[2].number;
             //mean reserve density class 1:
             simdata[m,d].cohort_e[0]:=pop.sizeclass[0].mean_e;
             //mean reserve density class 2:
             simdata[m,d].cohort_e[1]:=pop.sizeclass[1].mean_e;
             //mean reserve density class 3:
             simdata[m,d].cohort_e[2]:=pop.sizeclass[2].mean_e;
             //saves feeding rate of the population on actual day:
```

```
            simdata[m,d].popfeedingrate:=pop.feedingrate;
            //saves amount of food that was left ona actual day:
            simdata[m,d].foodleft:=env.foodleft[d];
         end;
     //delete the population
     pop.destroy;
     //updates progress bar:
     Output.Form_Output.Progressbar1.Position:=(m+1);
    end;
    //calculates population endpoints from simulation results:
    calculatepopendpoints;
    //shows population endpoints in diagram and table:
    Output.Form_Output.showpopresults (endpoint);
    //shows diagrams at the end of the simulation:
    Output.Form_Output.Show;
end;

{xxxxxxxxxxxxxxxxxxxxxxxxxxxxxxxxxxxxxxxxxxxxxxxxxxxxxxxxxxxxxxxxxxxxxxxxxxxxxxxxxx
              Single setting simulation
xxxxxxxxxxxxxxxxxxxxxxxxxxxxxxxxxxxxxxxxxxxxxxxxxxxxxxxxxxxxxxxxxxxxxxxxxxxxxxxxxxx}
procedure TForm_Ecosystem.runregularsimulation;
begin
  //sets lenth of array to save simulation results for each simulation run and each day:
  setlength (Simdata,eco.simnumber,eco.simtime);
  //creates an environment:
  env:=T_Environment.create;
  //runs simulation:
  simulationrun;
end;

{xxxxxxxxxxxxxxxxxxxxxxxxxxxxxxxxxxxxxxxxxxxxxxxxxxxxxxxxxxxxxxxxxxxxxxxxxxxxxxxxxx
              Read settings, clear everything
xxxxxxxxxxxxxxxxxxxxxxxxxxxxxxxxxxxxxxxxxxxxxxxxxxxxxxxxxxxxxxxxxxxxxxxxxxxxxxxxxxx}
procedure TForm_Ecosystem.readsettings;
var
simnr:integer;
begin
  case RG_CI.ItemIndex of
    0: simnr:=10;   //10 MC simulations
    1: simnr:=100;  //100 MC simulations
    2: simnr:=1000; //1000 MC simulations
  end;
  case RG_Simtype.ItemIndex of
    0:eco.simnumber:=1;
    1:eco.simnumber:=simnr;
  end;
  eco.simtime:=strtoint(Environment.Form_Environment.E_Simtime.text);
  //Sets progress bar:
   Output.Form_Output.ProgressBar1.Min:=0;
   Output.Form_Output.ProgressBar1.Max:=eco.simnumber;
  //Clears diagrams and other output:
  Output.Form_Output.cleareverything;
```

```
{xxxxxxxxxxxxxxxxxxxxxxxxxxxxxxxxxxxxxxxxxxxxxxxxxxxxxxxxxxxxxxxxxxxxxxxxxxxxxxxxxx
              Calculate endpoints of model simulations
xxxxxxxxxxxxxxxxxxxxxxxxxxxxxxxxxxxxxxxxxxxxxxxxxxxxxxxxxxxxxxxxxxxxxxxxxxxxxxxxxxxx}
procedure TForm_Ecosystem.calculatepopendpoints;
var
m,d,c:integer;
SumSq, Conf, t, pop1, pop2: double;
begin
  setlength (endpoint.popresult,eco.simtime);

  //calculates every day endpoints:
  for d := 0 to eco.simtime - 1 do
  begin
      //(re-)sets endpoints before calculating value for each day:
      setlength (endpoint.popresult[d].mean_abundance, 4);
      setlength (endpoint.popresult[d].lower_abundance, 4);
      setlength (endpoint.popresult[d].upper_abundance, 4);
      setlength (endpoint.popresult[d].e_sizeclass,3);
      endpoint.popresult[d].popfeedingrate_min:=100000000000;
      endpoint.popresult[d].popfeedingrate_mean:=0;
      endpoint.popresult[d].popfeedingrate_max:=0;
      endpoint.popresult[d].extprob:=0;

      //sets means in class c=0:
      for c := 0 to 3 do
       begin
          //c=0: total abundance; c=1-C=3: Classes 1-3
          endpoint.popresult[d].mean_abundance[c]:=0;
          if c<3 then endpoint.popresult[d].e_sizeclass[c]:=0;
       end;

      //runs through Monte-Carlo simulations for calculating simulation means:
      for m := 0 to eco.simnumber - 1 do
      begin
          //sum and range of population feeding rate:
          endpoint.popresult[d].popfeedingrate_mean:=endpoint.popresult[d].popfeedingrate_mean+simdata[m,d].popfeedingrate;
          if simdata[m,d].popfeedingrate < endpoint.popresult[d].popfeedingrate_min
            then endpoint.popresult[d].popfeedingrate_min:= simdata[m,d].popfeedingrate;
          if simdata[m,d].popfeedingrate > endpoint.popresult[d].popfeedingrate_max
            then endpoint.popresult[d].popfeedingrate_max:= simdata[m,d].popfeedingrate;

          //sums up population measures per class
          for c := 0 to 3 do
          begin
            //abundances:
            endpoint.popresult[d].mean_abundance[c]:=endpoint.popresult[d].mean_abundance[c]+simdata[m,d].cohort_abundance[c];
            //reserve densities:
            if c<3 then endpoint.popresult[d].e_sizeclass[c]:=endpoint.popresult[d].e_sizeclass[c]+simdata[m,d].cohort_e[c];
          end;

          //summs up number of populations that went extinct:
```

```
          if simdata[m,d].cohort_abundance[0]<=0 then  endpoint.popresult[d].extprob:=endpoint.popresult[d].extprob+1;
       end;

       //calculates mean values from sums:
        for c := 0 to 3 do
          begin
            endpoint.popresult[d].mean_abundance[c]:=endpoint.popresult[d].mean_abundance[c]/eco.simnumber;
            if c<3 then endpoint.popresult[d].e_sizeclass[c]:=endpoint.popresult[d].e_sizeclass[c]/eco.simnumber;;
          end;
        endpoint.popresult[d].popfeedingrate_mean:=endpoint.popresult[d].popfeedingrate_mean/eco.simnumber;

       //calculates extinction probability:
       endpoint.popresult[d].extprob:=endpoint.popresult[d].extprob/eco.simnumber;

      //t-statistics for calculating 95% confidence intervals:
      case RG_CI.ItemIndex of
          0: t:=1.812; //10 MC simulations
          1: t:=1.66;  //100 MC simulations
          2: t:=1.64;  //1000 MC simulations
      end;

      //runs through classes to calculate confidence intervals:
      for c:= 0 to 3 do
      begin
        //c=0: total abundance; c=1 to c=3: Classes 1-3
        SumSq:=0;
        //runs through MC simulations:
        for m := 0 to eco.simnumber - 1 do
          begin
            //Calculates sums of squared deviations:
            SumSq := SumSq + Sqr(simdata[m,d].cohort_abundance[c] - endpoint.popresult[d].mean_abundance[c]);
        end;
        //if running Monte-Carlo simulations (n>1):
        if RG_simtype.ItemIndex>0 then
        begin
          //calculates standard error of mean:
          s:=power(SumSq / (eco.simnumber*(eco.simnumber-1)),(1/2));
          //calculates confidence intervals:
          Conf:= t*s;
        end
        //if running single simulations:
        else
        Conf:=0;
        //calculates upper and lower confidence limits for abundance:
        endpoint.popresult[d].lower_abundance[c]:=endpoint.popresult[d].mean_abundance[c]-Conf;
        endpoint.popresult[d].upper_abundance[c]:=endpoint.popresult[d].mean_abundance[c]+Conf;
      end;
  end;
end;
end.
```

```
{::::::::::::::::::::::::::::::::::::::::::::::::::::::::::::::::::::::::::::::::::::::::::::::::::::::::::::::::::::::::::::::::::::::::::
:::::::::::::::::::::::::::::::::::::::::::::::::::::::::::::::::::::::::::::::::::::::::::::::::::::::::::::::::::::::::::::::::::::::::::
      This unit handles information on environmental settings
:::::::::::::::::::::::::::::::::::::::::::::::::::::::::::::::::::::::::::::::::::::::::::::::::::::::::::::::::::::::::::::::::::::::::::
::::::::::::::::::::::::::::::::::::::::::::::::::::::::::::::::::::::::::::::::::::::::::::::::::::::::::::::::::::::::::::::::::::::::::}
unit Environment;

interface

uses
  Windows, Messages, SysUtils, Variants, Classes, Graphics, Controls, Forms,
  Dialogs, StdCtrls, ExtCtrls,
  toxicant, LoadEnvData;

//---Declarations for environment settings----------------------------------------------------
type
 T_Environment = class
   //Volume [ml] of the environment
   volume:                                integer;
   //day of simulation
   day:                                   integer;
   //Total amount of food [mgC per enviroment and day]
   foodtotal:                             array of double;
   //Total amount of food [mgC per enviroment and day] + leftovers from previous day
   foodavailability:                      array of double;
   //Total amount of food [mgC per enviroment and day] - population feeding rate
   foodleft:                              array of double;
   //Food data as read from file
   fooddata:                              T_fooddata;

   constructor create;
   procedure readfood;
 end;

//---declarations of TForm-------------------------------------------------------------------
type
  TForm_Environment = class(TForm)
    P_food:                               TPanel;
    RG_food, RG_foodcalculation:          TRadioGroup;
    E_fooddensity, E_volume, E_Simtime:   TLabeledEdit;
    procedure RG_foodcalculationClick     (Sender: TObject);
  end;

var
  Form_Environment: TForm_Environment;

implementation
{$R *.dfm}

uses LoadData;
```

```
{xxxxxxxxxxxxxxxxxxxxxxxxxxxxxxxxxxxxxxxxxxxxxxxxxxxxxxxxxxxxxxxxxxxxxxxxxxxxxxxxxx
       Selection food calculation
xxxxxxxxxxxxxxxxxxxxxxxxxxxxxxxxxxxxxxxxxxxxxxxxxxxxxxxxxxxxxxxxxxxxxxxxxxxxxxxxxxxx}
procedure TForm_Environment.RG_foodcalculationClick(Sender: TObject);
begin
   case RG_foodcalculation.ItemIndex of
     0: begin E_fooddensity.EditLabel.Caption:='Food denstiy [mgC/ml]'; E_fooddensity.text:=floattostr(0.001); end;
     1: begin E_fooddensity.EditLabel.Caption:='Food total [mgC]'; E_fooddensity.text:=floattostr(1); end;
   end;
end;

{ T_Environment }
{xxxxxxxxxxxxxxxxxxxxxxxxxxxxxxxxxxxxxxxxxxxxxxxxxxxxxxxxxxxxxxxxxxxxxxxxxxxxxxxxxx
              Create environment and read environmental settings
xxxxxxxxxxxxxxxxxxxxxxxxxxxxxxxxxxxxxxxxxxxxxxxxxxxxxxxxxxxxxxxxxxxxxxxxxxxxxxxxxxxx}
constructor T_Environment.create;
begin
   //Sets ecosystem volume [ml] or dry weight [mg]:
   volume:=strtoint(Form_Environment.E_volume.text);
   //Sets amount of food in environment [mgC] for each day:
   readfood;
end;

{xxxxxxxxxxxxxxxxxxxxxxxxxxxxxxxxxxxxxxxxxxxxxxxxxxxxxxxxxxxxxxxxxxxxxxxxxxxxxxxxxx
              Read food settings
xxxxxxxxxxxxxxxxxxxxxxxxxxxxxxxxxxxxxxxxxxxxxxxxxxxxxxxxxxxxxxxxxxxxxxxxxxxxxxxxxxxx}
procedure T_Environment.readfood;
var
d, simtime:integer;
food:double;
begin
   //sets simulation time:
   simtime:=strtoint(Form_Environment.E_Simtime.Text);
   //sets length of environment arrays
   setlength (foodtotal,simtime);
   setlength (foodavailability,simtime);
   setlength (foodleft,simtime);

   //Sets food density
    case Form_Environment.RG_foodcalculation.ItemIndex of
     0: food:=strtofloat(Form_Environment.E_Fooddensity.Text)*volume;
     1: food:=strtofloat(Form_Environment.E_Fooddensity.Text);
    end;

   case Form_Environment.RG_food.ItemIndex of
    //if food given every day
    0: begin
         for d := 0 to simtime - 1 do
          begin
             foodtotal[d]:=food;
          end;
       end;
```

```
    //if food given in a certain scenario read from csv-file:
    1: begin
          //reads food seetings from file data array:
          fooddata:=T_Fooddata.create;
          //saves food setting as read from file in food array
          for d := 0 to simtime - 1 do
          begin
              foodtotal[d]:= fooddata.foodamount [d];
          end;
       end;
   end;
end;
end.
```

```
{:::::::::::::::::::::::::::::::::::::::::::::::::::::::::::::::::::::::::::::::::::::::::::::::::::::::::::::::::::::::::::::::::::::::
::::::::::::::::::::::::::::::::::::::::::::::::::::::::::::::::::::::::::::::::::::::::::::::::::::::::::::::::::::::::::::::::::::::::
      In this unit population level processes are calculated
::::::::::::::::::::::::::::::::::::::::::::::::::::::::::::::::::::::::::::::::::::::::::::::::::::::::::::::::::::::::::::::::::::::::
:::::::::::::::::::::::::::::::::::::::::::::::::::::::::::::::::::::::::::::::::::::::::::::::::::::::::::::::::::::::::::::::::::::::}
unit Population;

interface

uses
  Windows, Messages, SysUtils, Variants, Classes, Graphics, Controls, Forms,
  Dialogs, Contnrs, math, StdCtrls, ExtCtrls,
  Individual, Environment, Toxicant;

//---declarations of the population------------------------------------------------------
//record saves results of population feeding rates
type
   T_feeding = record
      //Amount of food available to population [mgC]
      availablefood:                     double;
      //potential feeding rate of population [mgC/h]
      potpopfeeding:                     double;
      //realized feeding rate of population  [mgC/h]
      realpopfeeding:                    double;
   end;

//record saves results of population demography:
type
   T_sizeclass = record
      //number of individuals in size class
      number:                            integer;
      //mean reserve density in size class
      mean_e:                            double;
   end;

//defines population class:
type
  T_population = class(tobject)
      //List of Individuals in the Population
      poplist:                           TObjectlist;
      //Individuals in the population with properties of The class T_individual
      ind:                               T_individual;
      //total abundance of individuals in startpopulation
      initialabundance:                  integer;
      //embryo information for creating new individual
      embryo_e, embryo_L, embryo_UH:     double;
      //size distriution: number in size classes;
      sizeclass:                         array of T_sizeclass;
      //realized feeding rate of the population [mgC/d]
      feedingrate:                       double;
      //saves results of potential and realized feeding per hour
      feeding:                           array of T_feeding;
```

```
      //body size or minimum age of individual in start population
      value1:                            double;
      //body size standard deviation or maximum age in start population
      value2:                            double;
      //adult number needed for initialization of start populationn
      adult_nr:                          integer;

      //creates a population
      constructor create;
      //deletes a population
      destructor destroy; override; //override defines a method that replaces a virtual parent class method
      //run through individuals in a given population
      procedure runthrough (env:T_Environment);
      //calculates feeding rate of population
      procedure calculatefeedingrate (env:T_Environment);
    end;

//---declarations of TForm-----------------------------------------------------------
type
  TForm_Population = class(TForm)
    E_class1_nr, E_class1_sizeclass,
    E_class2_nr, E_class3_nr,
    E_class3_sizeclass, E_Iterations:    TLabeledEdit;
    E_class1_size, E_class2_size,
    E_class3_size, E_class1_SD,
    E_class2_SD, E_class3_SD:            TEdit;
    L_number, L_size, L_sizeclass1,
    L_sizeclass3, Label1, Label2, L_SD:  TLabel;
    P_Startpop, Panel1:                  TPanel;
    RG_StartPop:                         TRadioGroup;
    procedure RG_StartPopClick           (Sender: TObject);

  public
    env:T_Environment;
  end;

var
  Form_Population: TForm_Population;

implementation
{$R *.dfm}
{ t_population }
{xxxxxxxxxxxxxxxxxxxxxxxxxxxxxxxxxxxxxxxxxxxxxxxxxxxxxxxxxxxxxxxxxxxxxxxxxxxxxxxxxxx
            Run through a population for each day of the simulation
xxxxxxxxxxxxxxxxxxxxxxxxxxxxxxxxxxxxxxxxxxxxxxxxxxxxxxxxxxxxxxxxxxxxxxxxxxxxxxxxxxxxx}
procedure T_population.runthrough (env:T_Environment);
var
i,n,s:integer;
size:double;
begin
```

```
//goes through population to let individuals sense their environment:
  for i := poplist.Count - 1 downto 0 do
  begin
     //defines individual as item (i) in the list:
     ind:=poplist[i] as T_individual;
     //individual senses population density:
     ind.density:=poplist.Count/env.volume;
     //sensing density has consequences for life history parameters according to hypotheses in the paper:
     ind.hypotheses;
  end;

  //calculates feeding of population and individual at actual day:
  calculatefeedingrate (env);
  //goes through population at actual day to stuggle for life:
  for i := poplist.Count - 1 downto 0 do
  begin
     //Defines individual as item (i) in the list:
     ind:=poplist[i] as T_individual;
     //individual has to struggle for life:
     ind.struggleforlife;
     //remove individual from population if dead
     if ind.alive=false then poplist.Remove(ind);
  end;

  //add new born individuals to poplist
   for i := poplist.Count - 1 downto 0 do
   begin
     //defines individual as item (i) in the list:
     ind:=poplist[i] as T_individual;
     //a mother releases offspring when ebryo reaches maturity at birth new individuals are created and added to population list:
     if ind.brood.b_UH >= ind.UBH then
     begin
        //inherits mother brood information to offspring:
         embryo_e:=ind.brood.b_e;
         embryo_L:=ind.brood.b_L;
         embryo_UH:=ind.brood.b_UH;
         for n := 0 to ind.R-1 do
          begin
             //creates new individual
             ind:=t_individual.create (embryo_e, embryo_L, embryo_UH);
             //adds it to the population:
             poplist.Add(ind);
          end;
     end;
   end;

  //recording of size class results:
  for s := 0 to length(sizeclass)-1 do
    begin
      sizeclass[s].number:=0;
      sizeclass[s].mean_e:=0;
    end;
```

```
  //goes through a population:
  for i := poplist.Count - 1 downto 0 do
  begin
    ind:=poplist[i] as T_individual;
    //assign individual to first class:
    if ind.L/ind.dm < strtofloat(Form_Population.E_class1_sizeclass.text)then s:=0;
    //or assign individual to second class:
    if (ind.L/ind.dm >= strtofloat(Form_Population.E_class1_sizeclass.text))
        and (ind.L/ind.dm < strtofloat(Form_Population.E_class3_sizeclass.text)) then s:=1;
    //or assign individual to third class:
    if ind.L/ind.dm >= strtofloat(Form_Population.E_class3_sizeclass.text) then s:=2;
    //add individual to respective class
    inc(sizeclass[s].number);
    //add resderve density to respective class for calculation of mean
    sizeclass[s].mean_e:=sizeclass[s].mean_e+ind.e;
  end;

  //reserve density for calculation of average reserves per class:
  for s := 0 to length(sizeclass)-1 do
  begin
    if sizeclass[s].number >0 then sizeclass[s].mean_e:=sizeclass[s].mean_e/sizeclass[s].number
    else sizeclass[s].mean_e:=0;
  end;
end;

{xxxxxxxxxxxxxxxxxxxxxxxxxxxxxxxxxxxxxxxxxxxxxxxxxxxxxxxxxxxxxxxxxxxxxxxxxxxxxxxxxxx
            Creates an population at start of the simulation run
xxxxxxxxxxxxxxxxxxxxxxxxxxxxxxxxxxxxxxxxxxxxxxxxxxxxxxxxxxxxxxxxxxxxxxxxxxxxxxxxxxxx}
constructor t_population.create;
var
i:integer;
begin
  inherited;
 //Execute the (T_Population) constructor:
  poplist:=tobjectlist.Create(true);
 //---creates class1 individuals at simulation start----------------------------
  //abundance of individual at simulation start:
  initialabundance:=strtoint(Form_Population.E_class1_nr.text);
  //mean body size of individual at simulation start:
  value1:=strtofloat(Form_Population.E_class1_size.text);
  value2:=strtofloat(Form_Population.E_class1_SD.text);
  //counter for adult number
  adult_nr:=0;
  //creates individual neonates:
  for i := 0 to initialabundance - 1 do
  begin
      ind:=t_individual.create (value1, value2, adult_nr);
      poplist.Add(ind);
  end;
```

```
//---creates class2 individuals at simulation start----------------------------
  //abundance of individual at simulation start:
  initialabundance:=strtoint(Form_Population.E_class2_nr.text);
  //mean body size of individual at simulation start:
  value1:=strtofloat(Form_Population.E_class2_size.text);
  value2:=strtofloat(Form_Population.E_class2_SD.text);
  //creates individual juvenile:
  for i := 0 to initialabundance - 1 do
  begin
      ind:=t_individual.create (value1, value2, adult_nr);
      poplist.Add(ind);
  end;

 //---creates class3 individuals at simulation start----------------------------
  //abundance of individual at simulation start:
  initialabundance:=strtoint(Form_Population.E_class3_nr.text);
  //mean body size of individual at simulation start:
  value1:=strtofloat(Form_Population.E_class3_size.text);
  value2:=strtofloat(Form_Population.E_class3_SD.text);
  //creates individual adults:
  for i := 0 to initialabundance - 1 do
  begin
      ind:=t_individual.create (value1, value2, adult_nr);
      poplist.Add(ind);
      inc(adult_nr);
  end;

  setlength (feeding,24);
  setlength(sizeclass,3);
end;

{xxxxxxxxxxxxxxxxxxxxxxxxxxxxxxxxxxxxxxxxxxxxxxxxxxxxxxxxxxxxxxxxxxxxxxxxxxxxxxxxxxx
            Calculates potential and realized feeding of a population
xxxxxxxxxxxxxxxxxxxxxxxxxxxxxxxxxxxxxxxxxxxxxxxxxxxxxxxxxxxxxxxxxxxxxxxxxxxxxxxxxxx}
procedure T_population.calculatefeedingrate;
var
h,i,s,iter:integer;
begin
  iter:=strtoint(Form_Population.E_Iterations.Text);
  //resets population feeding rates:
  feedingrate:=0;
   for h := 0 to 24 - 1 do
    begin
      feeding[h].potpopfeeding:=0;
      feeding[h].realpopfeeding:=0;
    end;

  //Calculates daily feeding of population and individual
  for h := 0 to 24 - 1 do
    begin
     //sets available food to the amount left from previous time step:
```

```
 if h=0 then feeding[h].availablefood:=env.foodavailability[env.day]
     else feeding[h].availablefood:=feeding[h-1].availablefood;
 if feeding[h].availablefood >0 then
 begin
//go though Population to calculate potential feeding rate assuming unlimited food:
     for i := poplist.Count - 1 downto 0 do
       begin
         ind:=poplist[i] as T_individual;
         //resets the individuals ingestion rate:
         ind.ir[h]:=0;
         //calculates feeding of individual:
         feeding[h].potpopfeeding:=feeding[h].potpopfeeding+(ind.fx*ind.ill*power(ind.L,2)*
          (feeding[h].availablefood/env.volume)/(ind.xk+(feeding[h].availablefood/env.volume)));
       end;

//if enough food is available:
     if feeding[h].potpopfeeding <= feeding[h].availablefood then
       begin
         //calculates ingestion rate [mgC/h] of the individual:
         for i := poplist.Count - 1 downto 0 do
           begin
             ind:=poplist[i] as T_individual;
             ind.ir[h]:=(ind.fx*ind.ill*power(ind.L,2)*
                (feeding[h].availablefood/env.volume)/(ind.xk+(feeding[h].availablefood/env.volume)));
             ind.fd[h]:=feeding[h].availablefood/env.volume;
          end;
          //realized feeding of population equals potential feeding:
          feeding[h].realpopfeeding:=feeding[h].potpopfeeding;
          feeding[h].availablefood:=feeding[h].availablefood-feeding[h].potpopfeeding;
       end
//if population likes to eat more than the amount available, then reduce time interval: h/iter
     else
         begin
          //calculate feeding for shorter timesteps by means of iterations
          for s := 0 to iter- 1 do
            begin
               //go through population and calculate individual feeding rate for short time inteval
               for i := poplist.Count - 1 downto 0 do
                begin
                     ind:=poplist[i] as T_individual;
                     if feeding[h].availablefood >0 then
                     begin
                       //calculates ingestion rate of the individual:
                       ind.ir[h]:=ind.ir[h]+((ind.fx*ind.ill*power(ind.L,2))*
                                (feeding[h].availablefood/env.volume)/(ind.xk+(feeding[h].availablefood/env.volume))/iter);
                       //calculates population feeding rate:
                       feeding[h].realpopfeeding:=feeding[h].realpopfeeding+((ind.fx*ind.ill*power(ind.L,2))*
                                 feeding[h].availablefood/env.volume)/(ind.xk+(feeding[h].availablefood/env.volume))/iter);
                       //calculates actual food availability:
                       feeding[h].availablefood:=feeding[h].availablefood-((ind.fx*ind.ill*power(ind.L,2))*
                                (feeding[h].availablefood/env.volume)/(ind.xk+(feeding[h].availablefood/env.volume))/iter);
                     end;
```

```
                        ind.fd[h]:=feeding[h].availablefood/env.volume;
                    end;
                end;
                if feeding[h].availablefood < 0 then feeding[h].availablefood:=0;
             end;
      end;
    end;

  //Sums up hourly realized feeding rates to daily feeding rate
    for h := 0 to 24 - 1 do
    begin
       feedingrate:=feedingrate+feeding[h].realpopfeeding;
    end;
end;

{xxxxxxxxxxxxxxxxxxxxxxxxxxxxxxxxxxxxxxxxxxxxxxxxxxxxxxxxxxxxxxxxxxxxxxxxxxxxxxxxxx
            Destroy a population at the end of the simulation run
xxxxxxxxxxxxxxxxxxxxxxxxxxxxxxxxxxxxxxxxxxxxxxxxxxxxxxxxxxxxxxxxxxxxxxxxxxxxxxxxxxx}
destructor t_population.destroy;
begin
    poplist.Free;
    inherited;
end;

{xxxxxxxxxxxxxxxxxxxxxxxxxxxxxxxxxxxxxxxxxxxxxxxxxxxxxxxxxxxxxxxxxxxxxxxxxxxxxxxxxx
            Change start population settings
xxxxxxxxxxxxxxxxxxxxxxxxxxxxxxxxxxxxxxxxxxxxxxxxxxxxxxxxxxxxxxxxxxxxxxxxxxxxxxxxxxx}
procedure TForm_Population.RG_StartPopClick(Sender: TObject);
begin
case RG_StartPop.ItemIndex of
    0:begin
        L_size.Caption:='Size [mm]';  E_class1_size.Text:=floattostr(1);  E_class2_size.Text:=floattostr(1.6);
            E_class3_size.Text:=floattostr(3.2);
        L_SD.Caption:='SD';           E_class1_SD.Text:=floattostr(0.1);  E_class2_SD.Text:=floattostr(0.1);
            E_class3_SD.Text:=floattostr(0.1);      end;
    1:begin
        L_size.Caption:='min age';  E_class1_size.Text:='1';  E_class2_size.Text:='14';  E_class3_size.Text:='21';
        L_SD.Caption:='max age';    E_class1_SD.Text:='1';    E_class2_SD.Text:='20';    E_class3_SD.Text:='28';
      end;
end;
end;
end.
```

```
{:::::::::::::::::::::::::::::::::::::::::::::::::::::::::::::::::::::::::::::::::::::::::::::::::::::::::::::::::::::::::::::::::::::::
::::::::::::::::::::::::::::::::::::::::::::::::::::::::::::::::::::::::::::::::::::::::::::::::::::::::::::::::::::::::::::::::::::::::
      In this unit individual level processes are computed
::::::::::::::::::::::::::::::::::::::::::::::::::::::::::::::::::::::::::::::::::::::::::::::::::::::::::::::::::::::::::::::::::::::::
:::::::::::::::::::::::::::::::::::::::::::::::::::::::::::::::::::::::::::::::::::::::::::::::::::::::::::::::::::::::::::::::::::::::}
unit Individual;

interface

uses
  Windows, Messages, SysUtils, Variants, Classes, Graphics, Controls, Forms, Dialogs, math, Vcl.StdCtrls, Vcl.ExtCtrls,
  Environment;

//Defines a brood within an individual:
type
   T_brood = record
        //structural length of an animal in brood
        b_L:                             double;
        //scaled reserve density of an animal in brood
        b_e:                             double;
        //scaled maturity of an animal in brood
        b_UH:                            double;
        //Scaled mobilisation flux in brood
        b_Sc:                            double;
   end;

//Defines a class of individuals:
  T_individual = Class(TObject)

    //Individual parameter values
        //energy investment ratio
        g, g0:                           double;
        //volume-specific cost for structure
        EG:                              double;
        //somatic maintenance rate coefficient
        km, km0:                         double;
        //energy conductance
        v:                               double;
        //allocation fraction to soma
        kappa, kappa0:                   double;
        //Reproduction efficiency
        kr, kr0:                         double;
        //maturity maintenance rate coefficient
        kj,kj0:                          double;
        //reproductive rate (broodsize)
        R:                               integer;
        //cost of an egg
        U0E, U0E0:                       double;
        //shape coefficient
        dm:                              double;
        //aging aceleration
        ha:                              double;
```

```
    //Aging stress coefficient
    sG:                                double;
    //threshold function parameter
    e_alpha:                           double;
    //threshold function parameter
    e_beta:                            double;
    //tolerance density for crowding stress function
    dT:                                double;
    //no-effect density for crowding stress function
    d0:                                double;
    //damage recovery
    e_kd:                              double;
    //scaled maturity at birth
    UBH:                               double;
    //scaled maturity at puberty
    UPH:                               double;
    //surface-area-specific filtration rate
    fx, fx0:                           double;
    //incipient limiting level
    ill:                               double;
    //half saturation constant
    xk:                                double;
    //filtration adaptation factor
    fa:                                double;
    //surface area specific maximum assimilation rate;
    pAm:                               double;
    //minimum assimilation efficiency;
    pxmin, pxmin0:                     double;
    //maximum assimilation efficiency;
    pxmax:                             double;
    //scaled reserve density threshold for adaptation
    e0:                                double;
    //tolerance scaled reserve density for adaptation
    eT:                                double;

//Individual state variables and intermediate outputs
    //status of individual
    alive:                             boolean;
    //scaled reserve level
    Ue:                                double;
    //scaled catabolic flux
    Sc:                                double;
    //scaled reserve density
    e:                                 double;
    //structural length
    L:                                 double;
    //change in structural length
    dL:                                double;
    //scaled maturity
    UH:                                double;
    //scaled reproduction buffer
    UR:                                double;
```

```
   //scaled functional response
   f:                                double;
   //aging acceleration
   q:                                double;
   //hazard rate
   h:                                double;
   //scaled damage
   e_D, e_Dmax:                      double;
   //survival probability
   s:                                double;
   //stress function
   sD:                               double;
   //assimilation efficiency;
   px:                               double;
   //surface area specific assimilation rate
   pA:                               double;
   //ingestion rate of daphnid based on population feeding rate [mgC/h] saved per h
   ir:                               array of double;
   //food available in the environment, needed for calculation of assimilation efficacy
   fd:                               array of double;

//expected survival of an individual: random number [0...1] assigned at birth
   rs:double;

//state variables of brood:
   brood:                            T_brood;
   breeding:                         boolean;

//sensing the environment (population density: number of individuals per ml)
   density:                          double;

 //creates an individual:
 constructor create (embryo_e, embryo_L, embryo_UH:double); overload;
 //second constructor for start population
 Constructor create(value1, value2:double; adult_nr:integer); overload;
 //destroys an individual:
 destructor destroy; override;
 //adaptation-related and crowding related processes
 procedure hypotheses;
 //sums up procedures the individual has to undergo each day
 procedure struggleforlife;
 //calculates the growth of the individual
 procedure growth;
 //calculates maturation and allocation of energy to reproduction
 procedure maturation;
 //calculates food uptake of individual
 procedure feeding;
 //calculates of brood size and brood developmental state:
 procedure reproduction;
 //calculates aging, hazard and survival
 procedure survival;
```

```
      //read parameters of individual
      procedure readparameters;
  end;

//---declarations of TForm------------------------------------------------------------
type
  TForm_Individual = class(TForm)
    E_e0,  E_ealpha, E_ebeta, E_EG,
    E_eT, E_faa, E_fx, E_ha, E_ill,
    E_kappa, E_kd, E_kj, E_km, E_kr,
    E_pam, E_pxmax, E_pxmin, E_sg,
    E_U0E, E_UBH, E_UPH, E_v,  E_xk:     TLabeledEdit;
    Label1,  Label2, Label3, Label4:     TLabel;
    Panel1, Panel2, Panel3, Panel4:      TPanel;
    RG_adaptation, RG_crowding:          TRadioGroup;
    CB_Size_age: TCheckBox;

  public
    env:                                 T_environment;
  end;

var
  Form_Individual:                       TForm_Individual;

implementation
{$R *.dfm}

{ t_individual }
{xxxxxxxxxxxxxxxxxxxxxxxxxxxxxxxxxxxxxxxxxxxxxxxxxxxxxxxxxxxxxxxxxxxxxxxxxxxxxxxxxx
    Individual life cycle processes: feeding, growth, reproduction and survival
xxxxxxxxxxxxxxxxxxxxxxxxxxxxxxxxxxxxxxxxxxxxxxxxxxxxxxxxxxxxxxxxxxxxxxxxxxxxxxxxxxx}
procedure T_individual.struggleforlife;
begin
   //individual feeds:
   feeding;
   //individual allocates energy to maintenance and growth
   growth;
   //individual allocates energy to maturation or reproduction buffer
   maturation;
   //brood size and embryo development state
   reproduction;
   //aging acceleration, hazard rate and survival
   survival;
end;

{xxxxxxxxxxxxxxxxxxxxxxxxxxxxxxxxxxxxxxxxxxxxxxxxxxxxxxxxxxxxxxxxxxxxxxxxxxxxxxxxxx
  Density and food dependent changes in parameters and intermediate variables
xxxxxxxxxxxxxxxxxxxxxxxxxxxxxxxxxxxxxxxxxxxxxxxxxxxxxxxxxxxxxxxxxxxxxxxxxxxxxxxxxxx}
procedure T_individual.hypotheses;
begin
  //---Hypothesis H1--------------------------------------------
  if Form_Individual.RG_adaptation.ItemIndex=1 then
```

```
begin
    //adaptation of filtration rate
    fx:=fx0*(1+(fa*power(L,2)*math.max(0,((1-e)-(1-e0)))));
end;

//---Hypothesis H2--------------------------------------------
if Form_Individual.RG_adaptation.ItemIndex=2 then
begin
    sD:=1+(1/eT)*math.Max(0, ((1-e)-(1-e0)));
    //adaptation of energy fraction allocated to soma
    kappa:=math.min(1,(kappa0*sD));
    g:=EG*v/(kappa*pam);
end;

//---Hypothesis H3--------------------------------------------
if Form_Individual.RG_adaptation.ItemIndex=3 then
begin
    //adaptation of energy fraction allocated to soma
    sD:=1+(1/eT)*math.Max(0, ((1-e)-(1-e0)));
    kappa:=math.min(1,(kappa0*sD));
    g:=EG*v/(kappa*pam);
    //adaptation of filtration rate
    fx:=fx0*(1+(fa*power(L,2)*math.max(0,((1-e)-(1-e0)))));
end;

//---Hypothesis H4--------------------------------------------
if Form_Individual.RG_crowding.ItemIndex=1 then
begin
    dT:=0.106;
    d0:=0.02;
    sD:=1+(1/dT)*math.Max(0, ((density)-d0));
    //assuming that crowding acts on filtration rate
    if Form_Individual.RG_adaptation.ItemIndex=0 then fx:=fx0/sD
    else fx:=fx/sD;
end;

//---Hypothesis H5--------------------------------------------
if Form_Individual.RG_crowding.ItemIndex=2 then
begin
//stress function for kM
   dT:=  2.05;
   d0:=  0.15;
   sD:=1+(1/dT)*math.Max(0, ((density)-d0));
   km:=km0*sD;

//stress function for kJ
   dT:=0.29;
   d0:=0.021;
   sD:=1+(1/dT)*math.Max(0, ((density)-d0));
   kj:=kj0*sD;
end;
```

```
  //---Hypothesis H6-------------------------------------------
  if Form_Individual.RG_crowding.ItemIndex=3 then
  begin
   //stress function for filtration rate
     dT:=0.95;
     d0:=0.37;
     sD:=1+(1/dT)*math.Max(0, ((density)-d0));
     if Form_Individual.RG_adaptation.ItemIndex=0 then fx:=fx0/sD
      else fx:=fx/sD;

   //stress function for costs for reproduction
     dT:=0.59;//0.7;
     d0:=0.021;//0.021;
     sD:=1+(1/dT)*math.Max(0, ((density)-d0));
     U0E:=U0E0*sD;
  end;
end;

{xxxxxxxxxxxxxxxxxxxxxxxxxxxxxxxxxxxxxxxxxxxxxxxxxxxxxxxxxxxxxxxxxxxxxxxxxxxxxxxxxx
               aging acceleration, hazard rate and survival
xxxxxxxxxxxxxxxxxxxxxxxxxxxxxxxxxxxxxxxxxxxxxxxxxxxxxxxxxxxxxxxxxxxxxxxxxxxxxxxxxxxx}
procedure T_individual.survival;
var
Fe: double;
begin
  //calculates aging acceleration:
  q:=q+ ((q* (power(L,3)/power((v/(g*km)),3)*sG) + ha) *e*( ((v/L)-(3*dL/L)))-((3*dL/L)*q));
  //calculates hazard rate:
  h:=h+(q-(3*dL/L)*h);
  //calculates maximum scaled damage:
  e_D:=e_D+e_kd*(((1-e))-e_D);
  if e_Dmax<e_D then e_Dmax:=e_D;
  //gives threshold for starvation
  if e_Dmax>0 then Fe:=1/(1+power((e_Dmax/e_alpha),-e_beta))
  else Fe:=0;
  //calculates survival probability:
  s:=(1-Fe)*exp(-h);
  //animal dead?
  if s<=rs then alive:=false;
end;

{xxxxxxxxxxxxxxxxxxxxxxxxxxxxxxxxxxxxxxxxxxxxxxxxxxxxxxxxxxxxxxxxxxxxxxxxxxxxxxxxxx
               food uptake by an individual
xxxxxxxxxxxxxxxxxxxxxxxxxxxxxxxxxxxxxxxxxxxxxxxxxxxxxxxxxxxxxxxxxxxxxxxxxxxxxxxxxxxx}
procedure T_individual.feeding;
var
h:integer;
begin
  //resets daily assimilation:
  Pa:=0;
  //calculates assimilation rate on the basis of realized feeding rate
    for h := 0 to 24 - 1 do
```

```
      begin
         //gives assimilation efficiency:
         px:=pxmax-((pxmax-pxmin)*(fd[h]/(xk+fd[h])));
        //calculates assimilation rate (cummulative per day):
        Pa:=Pa + (px*ir[h]);
      end;
   //calculates actual scaled functional response:
   f:=Pa/(pAm*power(L,2));
   //safety request for model discretization:
   if f>1 then f:=1;
end;

{xxxxxxxxxxxxxxxxxxxxxxxxxxxxxxxxxxxxxxxxxxxxxxxxxxxxxxxxxxxxxxxxxxxxxxxxxxxxxxxxxx
        allocation of energy to growth of an individual
xxxxxxxxxxxxxxxxxxxxxxxxxxxxxxxxxxxxxxxxxxxxxxxxxxxxxxxxxxxxxxxxxxxxxxxxxxxxxxxxxxx}
procedure T_individual.growth;
var
s, step:integer;
sl:double;
begin
//Sets number of iterations:
step:=100;
//resets daily growth:
dL:=0;
//growth in iterations:
for s := 0 to step-1 do
begin
    //calculates scaled body length:
    sl:=L/(v*e/(km*g));
    //for growth conditions calculates:
    if e>sl then
     begin
        //calculates actual scaled catabolic flux:
        Sc:= power (L,2) *((g*e)/(g+e))*(1+(km*L/v));
        //calculates daily increase in length, needed for computing aging
        dL:=dL+ (1 /(3*power(L,2)))*(((v/g)*Sc) - km*power(L,3))/step;
        //calculates growth in iterations
        L:=L+ (1 /(3*power(L,2)))*(((v/g)*Sc) - km*power(L,3))/step;
      end
     else
     //for non-growth conditions calculates:
     begin
        // calculates actual scaled catabolic flux when only paying maintenance costs
        Sc:=km*kappa*g*power (L,3)/v;
        //and change in length is 0:
        dL:=0;
     end;

    //updates actual scaled reserves:
    Ue:= Ue+((((f*power (L,2))-Sc))/step);
    //updates actual scaled reserve density:
    e:=v*Ue/power (L,3);
```

```
    //safety request for model discretization:
    if e>1 then e:=1;
end;
end;

{xxxxxxxxxxxxxxxxxxxxxxxxxxxxxxxxxxxxxxxxxxxxxxxxxxxxxxxxxxxxxxxxxxxxxxxxxxxxxxxxxxx
         allocation of energy to maturation or reproduction
xxxxxxxxxxxxxxxxxxxxxxxxxxxxxxxxxxxxxxxxxxxxxxxxxxxxxxxxxxxxxxxxxxxxxxxxxxxxxxxxxxxx}
procedure T_individual.maturation;
begin
//if above non-growth boundary spend reserve on maturation or reproduction buffer, otherwise reserve is spent on maintenance costs only
if e>f*(L/(v/(km*g))) then
     begin
     //if individual does not yet reach puberty, energy flux to maturation:
     if UH < UPH then UH:= UH + (((1-kappa)*Sc) - (kj*UH))
     //if individual reaches puberty, energy flux into reproductive buffer:
     else UR:=UR +(((1-kappa)*Sc) - (kj*UPH));
end;
end;

{xxxxxxxxxxxxxxxxxxxxxxxxxxxxxxxxxxxxxxxxxxxxxxxxxxxxxxxxxxxxxxxxxxxxxxxxxxxxxxxxxxx
               reproduction by an individual
xxxxxxxxxxxxxxxxxxxxxxxxxxxxxxxxxxxxxxxxxxxxxxxxxxxxxxxxxxxxxxxxxxxxxxxxxxxxxxxxxxxx}
procedure T_individual.reproduction;
var
i,d:integer;
x:double;
begin
//starts reproduction:
if UH >= UPH then
begin
 //if breeding, calculates growth and maturation of embryos:
   if breeding then
   begin
      //calculated scaled catabolic flux and growth of the embryo under the assumption that e does not change:
        brood.b_Sc:= power (brood.b_L,2) *((g0*brood.b_e)/(g0+brood.b_e))*(1+(km0*brood.b_L/v));
        brood.b_L:=brood.b_L+ (1 /(3*power(brood.b_L,2)))*(((v/g0)*brood.b_Sc) - km0*power(brood.b_L,3));
      //Calculates scaled maturity of embryo
      brood.b_UH:= brood.b_UH + (((1-kappa0)*brood.b_Sc) - (kj0*brood.b_UH));
   end;
 //if not breeding then initialize brood:
   if not breeding then
   begin
       //structural length at egg formation:
       brood.b_L:=0.0000001;
       //scaled reserved density of brood is assumed to be equal to mother scaled reserve density at egg formation
       brood.b_e:=e;
       //scaled maturity at egg formation:
       brood.b_UH:=0;
       //calculates brood size:
       R:=trunc(kr*UR/U0E);
       //resets reproduction buffer, but leaves the rest of buffer that could not be used to produce a full egg:
```

```
        UR:=(kr*UR/U0E)-trunc(kr*UR/U0E);
        //start breeding:
        breeding:=true;
    end;
  //if embryos reach scaled maturity at birth (UPB) then stop breeding:
    if brood.b_UH >= UBH then
    begin
       breeding:=false;
    end;
end;
end;

{xxxxxxxxxxxxxxxxxxxxxxxxxxxxxxxxxxxxxxxxxxxxxxxxxxxxxxxxxxxxxxxxxxxxxxxxxxxxxxxxxxx
               Create an individual at birth
xxxxxxxxxxxxxxxxxxxxxxxxxxxxxxxxxxxxxxxxxxxxxxxxxxxxxxxxxxxxxxxxxxxxxxxxxxxxxxxxxxxx}
constructor t_individual.create (embryo_e, embryo_L, embryo_UH:double);
begin
    inherited create;
    //structural length [mm] at birth:
    L:=embryo_L;
    //scaled maturity [t*mm^2] at birth UH (0)=0:
    UH:=embryo_UH;
    //this is where parameters are stored:
    readparameters;
    //scaled reserve density [-]:
    e:=embryo_e;
    //scaled reserve level [t L^2]:
    UE:=(e*power(L,3))/v;
    //status of individual after birth
    alive:=true;
end;

{xxxxxxxxxxxxxxxxxxxxxxxxxxxxxxxxxxxxxxxxxxxxxxxxxxxxxxxxxxxxxxxxxxxxxxxxxxxxxxxxxxx
              creates individuals at simulation start
xxxxxxxxxxxxxxxxxxxxxxxxxxxxxxxxxxxxxxxxxxxxxxxxxxxxxxxxxxxxxxxxxxxxxxxxxxxxxxxxxxxx}
constructor T_individual.create(value1, value2: double; adult_nr:integer);
var
uppersize, lowersize: double;
count,d, dmax,min,max: integer;
begin
   inherited create;
   //reads parameters:
   readparameters;
   //structural length at birth:
   L:=dm*0.9;
   //Scaled maturity at birth (neonate <24h):
   UH:=UBH;
   //scaled reserve density under culture conditions:
   e:=0.7;
   //Scaled reserve level:
   UE:=(e*power(L,3))/v;
```

```
 //-----life cycle until individual reaches size at simulation start---------------------
  if Form_Individual.CB_Size_age.checked then
  begin
   //random body size of individual within one standard deviations from mean:
       uppersize:=value1 + value1*1*value2;
       lowersize:=value1 - value1*1*value2;

       if value2 > 0 then
       begin
         repeat
           value1:=randG(value1, value2);
         until (value1<uppersize) or (value1>lowersize);
       end;
       //under culture conditions with f=0.7 daphnids will not get bigger than this:
       if value1>4 then value1:=4;

     //runs through life cycle until respective size:
       repeat
         //scaled functional response in culture conditions:
         f:=0.7;
         //individual allocates energy to maintenance and growth
         growth;
         //individual allocates energy to maturation or reproduction buffer
         maturation;
         //Brood size and embryo development state
         reproduction;
         //aging acceleration, hazard rate and survival
         survival;
         //experiment started with living individuals only:
        alive:=true;
      until L>=value1*dm;
  end

//-----life cycle until individual age at simulation start---------------------
  else
  begin
   min:=round(value1); max:=round(value2);
   dmax:=randomrange (min, max);
   //runs through time
   for d := 0 to dmax-1 do
     begin
      //scaled functional response in culture conditions:
       f:=0.7;
       //individual allocates energy to maintenance and growth
       growth;
       //individual allocates energy to maturation or reproduction buffer
       maturation;
       //Brood size and embryo development state
       reproduction;
       //aging acceleration, hazard rate and survival
       survival;
       //experiment started with living individuals only:
```

```
        alive:=true;
       end;
    end;

    //at start of the experiments only adult daphnids were used that are not going to release neonates on the same day:
    if (UH>UPH) and (adult_nr>0) then brood.b_UH:=randomrange(0,5)/1000
    else brood.b_UH:=0;
end;

{xxxxxxxxxxxxxxxxxxxxxxxxxxxxxxxxxxxxxxxxxxxxxxxxxxxxxxxxxxxxxxxxxxxxxxxxxxxxxxxxxx
               This is where parameters of the individual are stored
xxxxxxxxxxxxxxxxxxxxxxxxxxxxxxxxxxxxxxxxxxxxxxxxxxxxxxxxxxxxxxxxxxxxxxxxxxxxxxxxxxx}
procedure T_individual.readparameters;
var
x:double;
begin
    {Please note that parameters and equations for calculating body weight are not included
     in the population model. They were needed for model parameterization only}

    //shape coefficient, fixed as it is necessary to calculate size classes only
    dm:=0.54;
    //Somatic maintenance rate coefficient:
    km:=strtofloat(Form_Individual.E_km.Text);//1.599;
    //Energy conductance:
    v:=strtofloat(Form_Individual.E_v.Text);
    //Allocation fraction to soma:
    kappa:=strtofloat(Form_Individual.E_kappa.Text);//0.678;
    //Volume specific cost for structure
    EG:=strtofloat(Form_Individual.E_EG.Text);//0.001787
    //reproduction efficiency:
    kr:=strtofloat(Form_Individual.E_kr.Text);//0.95
    //maturity maintenance rate coefficient:
    kj:=strtofloat(Form_Individual.E_kj.Text);//0.969;
    //Scaled maturity at birth:
    UBH:=strtofloat(Form_Individual.E_UBH.Text);//0.012
    //Scaled maturity at puperty:
    UPH:=strtofloat(Form_Individual.E_UPH.Text);//0.1997;
    //Scaled reproduction buffer at birth
    UR:=0;
    //Cost of an egg:
    U0E:=strtofloat(Form_Individual.E_U0E.Text);//0.089;
    //initial reproductive output:
    R:=0;  breeding:=false; brood.b_UH:=0;
    //Weibull aging acceleration
    ha:=strtofloat(Form_Individual.E_ha.Text);//0.00029;
    //Gompertz stress coefficient
    sG:=strtofloat(Form_Individual.E_sg.Text);//0.5;
    //random number that determines individual survival probability
    rs:=random;
    //damage recovery
    e_kd:=strtofloat(Form_Individual.E_kd.Text);//0.09;
    //median of thresold distribution
```

```
    e_alpha:=strtofloat(Form_Individual.E_ealpha.Text);//0.41;
    //slope of threshold distribution
    e_beta:=strtofloat(Form_Individual.E_ebeta.Text);//4;
    //hazard rate, damages at birth
    h:=0;  e_D:=0; q:=0;
    //scaling factor of maximum feeding/filtration rate
    repeat
      x:=randg(1,0.231);
    until (x <1.3) or (x<0.7);
    fx:=x*strtofloat(Form_Individual.E_fx.Text);
    //half saturation constant
    xk:=strtofloat(Form_Individual.E_xk.Text);//0.0002;
    //incipient limiting level
    ill:=strtofloat(Form_Individual.E_ill.Text);//0.00037;
    //Threshold scaled reserve density:
    e0:=strtofloat(Form_Individual.E_e0.Text);//0.63;
    //Tolerance scaled reserve density for low food adaptation:
    eT:=strtofloat(Form_Individual.E_eT.Text);//0.139;
    //Scaled increase in filtration rate
    fa:=strtofloat(Form_Individual.E_faa.Text);//10.4;
    //maximum surface area specific assimilation rate;
    pAm:=strtofloat(Form_Individual.E_pam.Text);//0.0145;
    //minimum and maximum assimilation efficiency;
    pxmin:=strtofloat(Form_Individual.E_pxmin.Text);//0.58;
    pxmax:=strtofloat(Form_Individual.E_pxmax.Text);//0.95;
    //set length of ingestion rate array:
    setlength (ir,24);
    //set length of food availability array:
    setlength (fd,24);
    //calculates energy investment ratio g:
    g:=EG*v/(kappa*pam);
    //saves original parameters for adaptation and crowding processes:
    kappa0:=kappa; km0:=km; kj0:=kj; g0:=g;  fx0:=fx; pxmin0:=pxmin; kr0:=kr;  U0E0:=U0E;
end;

{xxxxxxxxxxxxxxxxxxxxxxxxxxxxxxxxxxxxxxxxxxxxxxxxxxxxxxxxxxxxxxxxxxxxxxxxxxxxxxxxxx
               destroys an individual at death
xxxxxxxxxxxxxxxxxxxxxxxxxxxxxxxxxxxxxxxxxxxxxxxxxxxxxxxxxxxxxxxxxxxxxxxxxxxxxxxxxxxxx}
destructor T_individual.destroy;
begin
  inherited;
end;
end.
```

```
{:::::::::::::::::::::::::::::::::::::::::::::::::::::::::::::::::::::::::::::::::::::::::::::::::::::::::::::::::::::::::::::::::::::::
:::::::::::::::::::::::::::::::::::::::::::::::::::::::::::::::::::::::::::::::::::::::::::::::::::::::::::::::::::::::::::::::::::::::
     In this unit model results are shown at the user interface
:::::::::::::::::::::::::::::::::::::::::::::::::::::::::::::::::::::::::::::::::::::::::::::::::::::::::::::::::::::::::::::::::::::::
::::::::::::::::::::::::::::::::::::::::::::::::::::::::::::::::::::::::::::::::::::::::::::::::::::::::::::::::::::::::::::::::::::::}
unit Output;

interface

uses
  Windows, Messages, SysUtils, Variants, Classes, Graphics, Controls, Forms,  Dialogs, ComCtrls, TeEngine, Series, ExtCtrls, TeeProcs,
  Chart, grids, Vcl.StdCtrls;

//---Endpoints calculated from data that is produced in simulation runs----------------------------
type
  T_popresult=record
    //Endpoints for total abundance of individuals
    mean_abundance, lower_abundance,
    upper_abundance:                     array of double;
    //Reserve density in cohorts as means of MC simulations
    e_sizeclass:                         array of double;
    //Extinction probability: ratio of populations that went extinct to number of Monte-Carlo simulations
    extprob:                             double;
    //Endpoints for food dynamics
    popfeedingrate_mean,
    popfeedingrate_min,
    popfeedingrate_max:                  double;
   end;

type
  T_endpoint=record
    //Array of population results as saved per day
    popresult:                           array of T_popresult;
  end;


//---declarations of TForm--------------------------------------------------------------
type
  TForm_Output = class(TForm)
    Chart_adults, Chart_feedingrate,
    Chart_juvenile, Chart_neonates,
    Chart_reserves, Chart_totalabundance:TChart;
    S_adult_e, S_adults_max,
    S_adults_mean, S_adults_min,
    S_feedingrate_max, S_feedingrate_mean,
    S_feedingrate_min, S_juvenile_e,
    S_juveniles_max, S_juveniles_mean,
    S_juveniles_min, S_neonate_e,
    S_neonates_max, S_neonates_mean,
    S_neonates_min, S_total_max,
    S_total_mean, S_total_min:           TLineSeries;
    S_data_class1, S_data_class2,
```

```
    S_data_class3, S_data_total:        TPointSeries;
    TS_Food, TS_Numbers, TS_Population: TTabSheet;
    SaveDialog1:                        TSaveDialog;
    SG_Numbers:                         TStringGrid;
    Memo1:                              TMemo;
    PC_Output:                          TPageControl;
    ProgressBar1:                       TProgressBar;


  public
  endpoint:                             T_endpoint;
  procedure showpopresults              (endpoint:T_endpoint);
  procedure cleareverything;
  procedure savepopresult;
  end;

var
    Form_Output: TForm_Output;

implementation
{$R *.dfm}
{ TForm_Output }
{xxxxxxxxxxxxxxxxxxxxxxxxxxxxxxxxxxxxxxxxxxxxxxxxxxxxxxxxxxxxxxxxxxxxxxxxxxxxxxxxxx
            Show model output in Graphs
xxxxxxxxxxxxxxxxxxxxxxxxxxxxxxxxxxxxxxxxxxxxxxxxxxxxxxxxxxxxxxxxxxxxxxxxxxxxxxxxxxxx}
procedure TForm_Output.showpopresults(endpoint:T_endpoint);
var
d,i:integer;
line, name:string;
begin
//sets string grid for dynamics in numbers:
SG_numbers.rowcount:=length(endpoint.popresult);
SG_numbers.Cells[0,0]:='Time [d]';
SG_numbers.Cells[1,0]:='Total mean';
SG_numbers.Cells[2,0]:='Total min';
SG_numbers.Cells[3,0]:='Total max';
SG_numbers.Cells[4,0]:='Class 1 mean';
SG_numbers.Cells[5,0]:='Class 1 min';
SG_numbers.Cells[6,0]:='Class 1 max';
SG_numbers.Cells[7,0]:='Class 2 mean';
SG_numbers.Cells[8,0]:='Class 2 min';
SG_numbers.Cells[9,0]:='Class 2 max';
SG_numbers.Cells[10,0]:='Class 3 mean';
SG_numbers.Cells[11,0]:='Class 3 min';
SG_numbers.Cells[12,0]:='Class 3 max';
SG_numbers.Cells[13,0]:='Extinction probability';

//shows result for each day:
for d := 0 to length(endpoint.popresult) - 1 do
begin
    //Graphs for population endpoints
    S_total_min.AddXY(d+1,endpoint.popresult[d].lower_abundance[0]);
```

```
    S_total_mean.AddXY(d+1,endpoint.popresult[d].mean_abundance[0]);
    S_total_max.AddXY(d+1,endpoint.popresult[d].upper_abundance[0]);
    S_neonates_min.AddXY(d+1,endpoint.popresult[d].lower_abundance[1]);
    S_neonates_mean.AddXY(d+1,endpoint.popresult[d].mean_abundance[1]);
    S_neonates_max.AddXY(d+1,endpoint.popresult[d].upper_abundance[1]);
    S_juveniles_min.AddXY(d+1,endpoint.popresult[d].lower_abundance[2]);
    S_juveniles_mean.AddXY(d+1,endpoint.popresult[d].mean_abundance[2]);
    S_juveniles_max.AddXY(d+1,endpoint.popresult[d].upper_abundance[2]);
    S_adults_min.AddXY(d+1,endpoint.popresult[d].lower_abundance[3]);
    S_adults_mean.AddXY(d+1,endpoint.popresult[d].mean_abundance[3]);
    S_adults_max.AddXY(d+1,endpoint.popresult[d].upper_abundance[3]);

    //sting grid for population endpoint
    SG_numbers.Cells[0,d+1]:=inttostr(d+1);
    SG_numbers.Cells[1,d+1]:=floattostr(endpoint.popresult[d].mean_abundance[0]);
    SG_numbers.Cells[2,d+1]:=floattostr(endpoint.popresult[d].lower_abundance[0]);
    SG_numbers.Cells[3,d+1]:=floattostr(endpoint.popresult[d].upper_abundance[0]);
    SG_numbers.Cells[4,d+1]:=floattostr(endpoint.popresult[d].mean_abundance[1]);
    SG_numbers.Cells[5,d+1]:=floattostr(endpoint.popresult[d].lower_abundance[1]);
    SG_numbers.Cells[6,d+1]:=floattostr(endpoint.popresult[d].upper_abundance[1]);
    SG_numbers.Cells[7,d+1]:=floattostr(endpoint.popresult[d].mean_abundance[2]);
    SG_numbers.Cells[8,d+1]:=floattostr(endpoint.popresult[d].lower_abundance[2]);
    SG_numbers.Cells[9,d+1]:=floattostr(endpoint.popresult[d].upper_abundance[2]);
    SG_numbers.Cells[10,d+1]:=floattostr(endpoint.popresult[d].mean_abundance[3]);
    SG_numbers.Cells[11,d+1]:=floattostr(endpoint.popresult[d].lower_abundance[3]);
    SG_numbers.Cells[12,d+1]:=floattostr(endpoint.popresult[d].upper_abundance[3]);
    SG_numbers.Cells[13,d+1]:=floattostr(endpoint.popresult[d].extprob);

    //Graphs for food dynamics
    S_feedingrate_min.AddXY(d,endpoint.popresult[d].popfeedingrate_min);
    S_feedingrate_mean.AddXY(d,endpoint.popresult[d].popfeedingrate_mean);
    S_feedingrate_max.AddXY(d,endpoint.popresult[d].popfeedingrate_max);

    //Graphs for scaled reserve density
    S_neonate_e.AddXY(d,endpoint.popresult[d].e_sizeclass[0]);
    S_juvenile_e.AddXY(d,endpoint.popresult[d].e_sizeclass[1]);
    S_adult_e.AddXY(d,endpoint.popresult[d].e_sizeclass[2]);

end;
 //writes string results into memo for saving results:
 for I := 0 to SG_numbers.rowcount-1 do
 begin
   line:=SG_numbers.Cells[0,i] +';'+ SG_numbers.Cells[1,i]+';'+ SG_numbers.Cells[2,i]+';'+ SG_numbers.Cells[3,i]
         +';'+ SG_numbers.Cells[4,i]+';'+ SG_numbers.Cells[5,i]+';'+ SG_numbers.Cells[6,i]
         +';'+ SG_numbers.Cells[7,i]+';'+ SG_numbers.Cells[8,i]+';'+ SG_numbers.Cells[9,i]
         +';'+ SG_numbers.Cells[10,i]+';'+ SG_numbers.Cells[11,i]+';'+ SG_numbers.Cells[12,i]
         +';'+ SG_numbers.Cells[13,i]  ;
   memo1.Lines.Add(line);
 end;
end;
```

```
{xxxxxxxxxxxxxxxxxxxxxxxxxxxxxxxxxxxxxxxxxxxxxxxxxxxxxxxxxxxxxxxxxxxxxxxxxxxxxxxxxxx
            Clear graphs string grid
xxxxxxxxxxxxxxxxxxxxxxxxxxxxxxxxxxxxxxxxxxxxxxxxxxxxxxxxxxxxxxxxxxxxxxxxxxxxxxxxxxxx}
procedure TForm_Output.cleareverything;
var
i:integer;
begin
   S_total_min.Clear;
   S_total_mean.Clear;
   S_total_max.Clear;
   S_feedingrate_min.Clear;
   S_feedingrate_mean.Clear;
   S_feedingrate_max.Clear;
   S_neonate_e.Clear;
   S_juvenile_e.Clear;
   S_adult_e.Clear;
   S_neonates_min.clear;
   S_neonates_mean.clear;
   S_neonates_max.clear;
   S_juveniles_min.clear;
   S_juveniles_mean.clear;
   S_juveniles_max.clear;
   S_adults_min.clear;
   S_adults_mean.clear;
   S_adults_max.clear;
   memo1.lines.clear;
   for i := 0 to SG_numbers.RowCount - 1 do SG_numbers.Rows[i].Clear;
end;

{xxxxxxxxxxxxxxxxxxxxxxxxxxxxxxxxxxxxxxxxxxxxxxxxxxxxxxxxxxxxxxxxxxxxxxxxxxxxxxxxxxx
            Save model output to csv file
xxxxxxxxxxxxxxxxxxxxxxxxxxxxxxxxxxxxxxxxxxxxxxxxxxxxxxxxxxxxxxxxxxxxxxxxxxxxxxxxxxxx}
procedure TForm_Output.savepopresult;
var
name:string;
begin
 savedialog1.Filter := 'CSV (Comma delimited) (*.csv)|*.CSV';
 name := savedialog1.FileName;
 If Copy(name,Pos('.',name),Length(name)-Pos('.',name)+1) <> '.csv' Then name := name + '.csv';
 if savedialog1.Execute then memo1.Lines.SaveToFile(savedialog1.filename);
end;
end.
```

```
{:::::::::::::::::::::::::::::::::::::::::::::::::::::::::::::::::::::::::::::::::::::::::::::::::::::::::::::::::::::::::::::::::::::::::::::
::::::::::::::::::::::::::::::::::::::::::::::::::::::::::::::::::::::::::::::::::::::::::::::::::::::::::::::::::::::::::::::::::::::::::::::
      In this unit population data is uploaded
::::::::::::::::::::::::::::::::::::::::::::::::::::::::::::::::::::::::::::::::::::::::::::::::::::::::::::::::::::::::::::::::::::::::::::::
:::::::::::::::::::::::::::::::::::::::::::::::::::::::::::::::::::::::::::::::::::::::::::::::::::::::::::::::::::::::::::::::::::::::::::::}
unit LoadData;

interface

uses
  Winapi.Windows, Winapi.Messages, System.SysUtils, System.Variants, System.Classes, Vcl.Graphics,
  Vcl.Controls, Vcl.Forms, Vcl.Dialogs, Vcl.StdCtrls,
  Environment, Population, Output;

//---Information on start population-------------------------------------------
type
    t_popdata=array of record
    //number per size class, mean size in class and standard deviation of size
      nrclass:                            integer; //array over class: class 1(0)...total(3)
      sizeinclass:                        double;  //array over class: class 1(0)...total(3)
      SDclass:                            double; //array over class: class 1(0)...total(3)
    //actual population test data
      day:                                array of integer; //array over time
      num:                                array of double;  //array over time
end;

//---population data storage----------------------------------------------------
type
    T_data=record  //array over treatments
      //species in test:
      species:                            string;
      //test medium: water (0) sediment (1)
      medium:                             integer;
      //volume of media [ml]
      vol:                                integer;
      //simulation time [d]
      simultime:                          integer;
      //amount of food [mg C]
      foodmass:                           double;
      //unit given as density [mg C/l]:(0) or total amount [mg C/population]:(1)
      foodunit:                           integer;
      //food given daily/scenrio/once in the beginning (0/1/3); scenario: load data from csv file
      foodscenario:                       integer;
      //Upper limit of size class1 and lower limit of class 3
      limclass1, limclass3:               double;
      //data for each size class
      popdata:                            t_popdata;
end;
```

```
//---declarations of TForm------------------------------------------------------
  TForm_Load = class(TForm)
    Memo1:          TMemo;
    OpenDialog1:    TOpenDialog;
  public
    { Public declarations }
    data:           T_data;
    procedure       loadpopdata;
    procedure       showloaddata;
  end;

var
  Form_Load: TForm_Load;

implementation

{$R *.dfm}

{xxxxxxxxxxxxxxxxxxxxxxxxxxxxxxxxxxxxxxxxxxxxxxxxxxxxxxxxxxxxxxxxxxxxxxxxxxxxxx
                       load population data from file
xxxxxxxxxxxxxxxxxxxxxxxxxxxxxxxxxxxxxxxxxxxxxxxxxxxxxxxxxxxxxxxxxxxxxxxxxxxxxxxx}
{procedure loads data from csv file that looks e.g. like:
Test;                                     Population
Species;                                  Daphnia magna
Water/Sediment (0/1);                     0
Volume of medium [ml];                    20000
Simulation time [d];                      80
Feeding mass [mgC/x];                     12
unit x: density or total (0/1);           0
daily/scenario/once (0/1/3);                            1
Initial number in Size class 1;           10
Mean length of SC 1 [mm];                 1
SD of SC 1  [mm];                         0,2
Initial number of Size class;             0
Mean length of SC 2 [mm];                 0
SD of SC 2  [mm];                         0
Initial number of Size class 3;           5
Mean length of SC 3 [mm];                 4,1
SD of SC 3  [mm];                         0,2
Size class 1 < [mm];                      1,3
Size class 3 >= [mm];                     2,6
Literature;                               Gergs et al. (2013)
Test Item;                                Control

                        day ;    size1;    day;     size2;  day    size3    day    total
                          0 ;     10;      0 ;      0;       0       5       0      15
                          3 ;     33;      3 ;      6;       3       5       3      38
                          .        .        .        .       .       .       .       .
                          .        .        .        .       .       .       .       .
                          .        .        .        .       .       .       .       . }
```

```
procedure TForm_Load.loadpopdata;
var
t,i,a,c:integer;
character:char;
counter, nr_conc:integer;
line, val, tab:string;
begin
  //empties memo:
  Memo1.Clear;
  //opens csv file:
  OpenDialog1.Title:='Load population data';
  if OpenDialog1.Execute then Memo1.Lines.Loadfromfile (OpenDialog1.FileName);
  //set length of data arrays
  setlength (data.popdata,4);
  //setlength (data.popdata[c],(memo1.lines.Count - 23))
    for c := 0 to 4-1 do
      begin
        setlength (data.popdata[c].day,(memo1.lines.Count - 23));
        setlength (data.popdata[c].num,(memo1.lines.Count - 23));
      end;

  t:=0;
  //loads data:
  for i := 0 to memo1.lines.Count - 1 do
  begin
    c:=0;
    //writes memo line into variable 'line'
    line:=memo1.lines[i];
    //empties the variable val:
    val:='';
    //reads tab stop string
    tab:=';';
    //sets row counter to 0
    counter:=0;
    //goes through lines:
    for a := 1 to length(line) do
    begin
      character:=line[a];
      //if the current character is ';' (marks end of a field in csv file) then do...
      if character=tab then //please note that tap stop character will depend on computer language settings
      begin
         if counter=1 then
         begin
            //ignore first line of file
            //second line: species information
            if (i=1) and (val<>'')then data.species:=val;
            //third line: medium type
            if (i=2) and (val<>'')then data.medium:=strtoint(val);
            //4th line: envirmonmental volume
            if (i=3) and (val<>'')then data.vol:=strtoint(val);
            //5th line: simulation time [d]
            if (i=4) and (val<>'')then data.simultime:=strtoint(val);
```

```
    //6th line: amount of food [mg C]
    if (i=5) and (val<>'')then data.foodmass:=strtofloat(val);
    //7th line: food density or total
    if (i=6) and (val<>'')then data.foodunit:=strtoint(val);
    //8th line: food given daily/scenario/once in the beginning
    if (i=7) and (val<>'')then data.foodscenario:=strtoint(val);
    //9th line: number in class 1
    if (i=8) and (val<>'')then data.popdata[1].nrclass :=strtoint(val);
    //10th line: size mean  in class 1
    if (i=9) and (val<>'')then data.popdata[1].sizeinclass :=strtofloat(val);
    //11th line: size standard deviation in class 1
    if (i=10)and (val<>'')then data.popdata[1].SDclass :=strtofloat(val);
    //12th line: number in class 2
    if (i=11) and (val<>'')then data.popdata[2].nrclass :=strtoint(val);
    //13th line: size mean  in class 2
    if (i=12) and (val<>'')then data.popdata[2].sizeinclass :=strtofloat(val);
    //14th line: size standard deviation in class 2
    if (i=13)and (val<>'')then data.popdata[2].SDclass :=strtofloat(val);
    //15th line: number in class 3
    if (i=14) and (val<>'')then data.popdata[3].nrclass :=strtoint(val);
    //16th line: size mean  in class 2
    if (i=15) and (val<>'')then data.popdata[3].sizeinclass :=strtofloat(val);
    //17th line: size standard deviation in class 3
    if (i=16)and (val<>'')then data.popdata[3].SDclass :=strtofloat(val);
    //18th line: upper limit of class 1
    if (i=17)and (val<>'')then data.limclass1:=strtofloat(val);
    //19th line: lower limit of class 3
    if (i=18)and (val<>'')then data.limclass3:=strtofloat(val);
    //ignore lines 20 - 23
end;

//23th line and higher: even numbers: numbers (ydata)
if (i>=23) and (val<>'') and (counter<8)then
begin
    //even column numbers: time [days]
    if ((counter/2)=trunc(counter/2)) then
    begin
      if val='x' then val:='1000000';
      data.popdata[c].day[t]:=strtoint(val);
    end
    //odd column numbers: numbers per size class (1-3) or total (4)
    else
    begin
        if val='x' then val:='0';
        data.popdata[c].num[t]:=strtofloat(val);
        //inc(t);
        inc(c);
    end;
end;

//increase column counter
inc(counter);
```

```
        //if a value is fixed then empty variable val
        val:='';
      end
      //merges single characters to a value (e.g. 1+2+3; --> 123)
      else val:=val+character;
    end;
    //row counter for popdata
    if (i>=23) then inc(t);
  end;
end;

{xxxxxxxxxxxxxxxxxxxxxxxxxxxxxxxxxxxxxxxxxxxxxxxxxxxxxxxxxxxxxxxxxxxxxxxxxxxxxxxx
                       shows population data
xxxxxxxxxxxxxxxxxxxxxxxxxxxxxxxxxxxxxxxxxxxxxxxxxxxxxxxxxxxxxxxxxxxxxxxxxxxxxxxxx}
procedure TForm_Load.showloaddata;
var
t:integer;
begin
   //clears data in graphs:
   output.Form_Output.S_data_class1.Clear;
   output.Form_Output.S_data_class2.Clear;
   output.Form_Output.S_data_class3.Clear;
   output.Form_Output.S_data_total.Clear;

   //sets environmental volume:
   Form_Environment.E_volume.text:=inttostr(data.vol);
   //sets simulation time:
   Form_Environment.E_Simtime.text:=inttostr(data.simultime);
   //sets amount of food:
   Form_Environment.E_fooddensity.text:=floattostr(data.foodmass);

   //sets food scenario
   case data.foodscenario of
     0: Form_Environment.RG_food.ItemIndex:=0;
     1: Form_Environment.RG_food.ItemIndex:=1;
     2: Form_Environment.RG_food.ItemIndex:=1;
   end;
   //sets initial population:
   Form_Population.E_class1_nr.text:=inttostr(data.popdata[1].nrclass);
        Form_Population.E_class1_size.text:=floattostr(data.popdata[1].sizeinclass);
            Form_Population.E_class1_SD.text:=floattostr(data.popdata[1].SDclass);
   Form_Population.E_class2_nr.text:=inttostr(data.popdata[2].nrclass);
        Form_Population.E_class2_size.text:=floattostr(data.popdata[2].sizeinclass);
            Form_Population.E_class2_SD.text:=floattostr(data.popdata[2].SDclass);
   Form_Population.E_class3_nr.text:=inttostr(data.popdata[3].nrclass);
        Form_Population.E_class3_size.text:=floattostr(data.popdata[3].sizeinclass);
            Form_Population.E_class3_SD.text:=floattostr(data.popdata[3].SDclass);

   //sets upper and lower limits of size classes:
   Form_Population.E_class1_sizeclass.text:=floattostr(data.limclass1);
   Form_Population.E_class3_sizeclass.text:=floattostr(data.limclass3);
```

```
   //shows data in graphs:
   output.Form_Output.Chart_neonates.Title.Caption:= 'Abundance fraction ≤' + floattostr (data.limclass1) +' mm';
   output.Form_Output.Chart_juvenile.Title.Caption:= 'Abundance fraction '  + floattostr (data.limclass1) + '-'+ floattostr
        (data.limclass3)  +' mm';
   output.Form_Output.Chart_adults.Title.Caption:=   'Abundance fraction >' + floattostr (data.limclass3) +' mm';

   for t := 0 to length(data.popdata[1].num)-1  do
   begin
       output.Form_Output.S_data_class1.AddXY(data.popdata[0].day[t],data.popdata[0].num[t]);
       output.Form_Output.S_data_class2.AddXY(data.popdata[0].day[t],data.popdata[1].num[t]);
       output.Form_Output.S_data_class3.AddXY(data.popdata[2].day[t],data.popdata[2].num[t]);
       output.Form_Output.S_data_total.AddXY(data.popdata[3].day[t],data.popdata[3].num[t]);
   end;
end;
end.
```

```
{:::::::::::::::::::::::::::::::::::::::::::::::::::::::::::::::::::::::::::::::::::::::::::::::::::::::::::::::::::::::::::::::::::::::::::
::::::::::::::::::::::::::::::::::::::::::::::::::::::::::::::::::::::::::::::::::::::::::::::::::::::::::::::::::::::::::::::::::::::::::::
      In this unit food input data is uploaded
::::::::::::::::::::::::::::::::::::::::::::::::::::::::::::::::::::::::::::::::::::::::::::::::::::::::::::::::::::::::::::::::::::::::::::
:::::::::::::::::::::::::::::::::::::::::::::::::::::::::::::::::::::::::::::::::::::::::::::::::::::::::::::::::::::::::::::::::::::::::::}
unit LoadEnvData;

interface

uses
  Winapi.Windows, Winapi.Messages, System.SysUtils, System.Variants, System.Classes, Vcl.Graphics,
  Vcl.Controls, Vcl.Forms, Vcl.Dialogs, Vcl.StdCtrls;

//---declarations of food data--------------------------------------------------------------------
type
    T_fooddata=class
    foodamount: array of double;
    constructor create;
end;

//---declarations of TForm--------------------------------------------------------------------
type
    TForm_LoadEnv = class(TForm)
    OpenDialog1: TOpenDialog;
    OpenDialog2: TOpenDialog;
    Memo1: TMemo;
    Memo2: TMemo;
  end;

var
  Form_LoadEnv: TForm_LoadEnv;

implementation
{$R *.dfm}
uses LoadData;
{xxxxxxxxxxxxxxxxxxxxxxxxxxxxxxxxxxxxxxxxxxxxxxxxxxxxxxxxxxxxxxxxxxxxxxxxxxxxxxx
                       load food data from file
xxxxxxxxxxxxxxxxxxxxxxxxxxxxxxxxxxxxxxxxxxxxxxxxxxxxxxxxxxxxxxxxxxxxxxxxxxxxxxxx}
{procedure loads food data from csv file that looks e.g. like:
Day;          Food conc. [mgC/l]; n.n.;
0;    6;
1;            0;
2;            0;
3;            6;
...}
constructor T_fooddata.create;
var
t,i,a:integer;
character:char;
counter, nr_conc:integer;
line, val, tab:string;
begin
```

```
  Form_LoadEnv.Memo1.Clear;
  Form_LoadEnv.OpenDialog1.Title:='Load food data';
  if Form_LoadEnv.OpenDialog1.Execute then Form_LoadEnv.Memo1.Lines.Loadfromfile (Form_LoadEnv.OpenDialog1.FileName);
  setlength(foodamount,Form_LoadEnv.memo1.lines.Count-1);
  t:=0;
  //starts data loading:
  for i := 0 to Form_LoadEnv.memo1.lines.Count - 1 do
  begin
    //writes memo line into variable 'line'
    line:=Form_LoadEnv.memo1.lines[i];
    //empties the variable val:
    val:='';
    //reads tab stop string
    tab:=';';
    //sets column counter to 0
    counter:=0;
    //goes through lines
    for a := 1 to length(line) do
    begin
      character:=line[a];
      //if the current character is ';' (tap stop in csv file) then do...
      if character=tab then //please note that tap stop character will depend on computer language settings
      begin
        //ignore first line
        //from second line: concentrations in second column, ignore first column
        if (i>0) and (val<>'')then
        begin
              if counter=1 then
             begin
                foodamount[t]:=strtofloat(val);
                inc(t);
             end;
        end;
        //increase column counter
        inc(counter);
        //empties variable val:
        val:='';
      end
      //merges single characters to a value (e.g. 1+2+3; --> 123)
      else val:=val+character;
    end;
  end;
end;
end.
```
